# Supplementary material for: Efficacy of Transarterial Chemoembolization Combined with Molecular Targeted Agents for Unresectable Hepatocellular Carcinoma: A Network Meta-Analysis
Source: Cancers (Basel). 2022 Jul 29;14(15):3710. doi: 10.3390/cancers14153710 (PMC9367476; doi:10.3390/cancers14153710)
Supplement: Supplementary file 1 [file cancers-14-03710-s001.zip › cancers-1784207-supplementary.pdf]

Supplementary Materials

Supplementary Material S1. Results of full search strategy of database.

Search strategy in EMBASE.		
No.	Query	Results
#34	#12 AND #14 AND #33	1771
#33	#17 OR #20 OR #23 OR #26 OR #29 OR #32	42587
#32	#30 OR #31	5018
#31	cabozantinib:ab,ti OR cometriq:ab,ti OR 'xl 184':ab,ti OR 'xl184 cpd':ab,ti OR 'xl-184':ab,ti OR 'bms 907351':ab,ti OR 'bms907351':ab,ti OR 'bms-907351':ab,ti	2124
#30	'cabozantinib'/exp	4867
#29	#27 OR #28	652
#28	catequentinib:ab,ti OR anlotinib:ab,ti OR al3818:ab,ti	500
#27	'catequentinib'/exp	615
#26	#24 OR #25	1742
#25	apatinib:ab,ti OR yn968d1:ab,ti OR 'yn-968d1':ab,ti OR rivoceranib:ab,ti	1337
#24	'rivoceranib'/exp	1634
#23	#21 OR #22	5016
#22	regorafenib:ab,ti OR stivarga:ab,ti OR 'bay 73-4506':ab,ti OR 'bay73-4506':ab,ti OR 'bay-73-4506':ab,ti	2792
#21	'regorafenib'/exp	4848
#20	#18 OR #19	3693
#19	lenvatinib:ab,ti OR lenvima:ab,ti OR 'e 7080':ab,ti OR 'e-7080':ab,ti OR 'er-203492-00':ab,ti OR e7080:ab,ti	2053

#18	'lenvatinib'/exp	3556
#17	#15 OR #16	33239
#16	sorafenib:ab,ti OR nexavar:ab,ti OR 'bay 43-9006':ab,ti OR 'bay 43 9006':ab,ti OR 'bay 439006':ab,ti OR 'bay-673472':ab,ti OR 'bay 673472':ab,ti OR 'bay 545-9085':ab,ti OR 'bay 545 9085':ab,ti OR 'bay 5459085':ab,ti OR 'bay-545-9085':ab,ti OR bay5459085:ab,ti	17356
#15	'sorafenib'/exp	32178
#14	'transarterial chemoembolization':ab,ti OR tace:ab,ti	13504
#13	'liver tumor'/exp	320254
#12	#9 OR #10 OR #11	357590
#11	'liver tumor'/exp	320254
#10	'liver cell carcinoma'/exp	179156
#9	'carcinoma, hepatocellular':ab,ti OR 'hepatocellular carcinomas':ab,ti OR 'carcinomas, hepatocellular':ab,ti OR 'liver cell carcinoma':ab,ti OR 'carcinoma, liver cell':ab,ti OR 'carcinomas, liver cell':ab,ti OR 'cell carcinoma, liver':ab,ti OR 'cell carcinomas, liver':ab,ti OR 'liver cell carcinomas':ab,ti OR 'hepatocellular carcinoma':ab,ti OR hepatoma:ab,ti OR hepatomas:ab,ti OR 'liver neoplasms':ab,ti OR 'neoplasms, hepatic':ab,ti OR 'neoplasms, liver':ab,ti OR 'liver neoplasm':ab,ti OR 'neoplasm, liver':ab,ti OR 'hepatic neoplasms':ab,ti OR 'hepatic neoplasm':ab,ti OR 'neoplasm, hepatic':ab,ti OR 'cancer of liver':ab,ti OR 'hepatocellular cancer':ab,ti OR 'cancers, hepatocellular':ab,ti OR 'hepatocellular cancers':ab,ti OR 'hepatic cancer':ab,ti OR 'cancer, hepatic':ab,ti OR 'cancers, hepatic':ab,ti OR 'hepatic cancers':ab,ti OR 'liver cancer':ab,ti OR 'cancer, liver':ab,ti OR 'cancers, liver':ab,ti OR 'liver cancers':ab,ti OR 'cancer of the liver':ab,ti OR 'cancer, hepatocellular':ab,ti	201225
#8	#3 AND #4 AND #7	1595
#7	#5 OR #6	33227

#6	sorafenib:ab,ti	17247
#5	'sorafenib'/exp	32178
#4	'transarterial chemoembolization':ab,ti OR tace:ab,ti	13504
#3	#1 OR #2	180707
#2	'carcinomas, hepatocellular':ab,ti OR 'hepatocellular carcinomas':ab,ti OR 'liver neoplasms':ab,ti	8323
#1	'liver cell carcinoma'/exp	179156

Search strategy in Cochrane.

ID	Search
#1	MeSH descriptor: [Carcinoma, Hepatocellular] explode all trees
#2	(Carcinomas, Hepatocellular):ti,ab,kw OR (Hepatocellular Carcinomas):ti,ab,kw OR (Hepatocellular Carcinoma):ti,ab,kw
#3	(Hepatoma):ti,ab,kw OR (Hepatomas):ti,ab,kw
#4	#1 or #2 or #3
#5	(TACE):ti,ab,kw OR (transarterial chemoembolization):ti,ab,kw
#6	MeSH descriptor: [Sorafenib] explode all trees
#7	#4 and #5 and #6
#8	MeSH descriptor: [Carcinoma, Hepatocellular] explode all trees
#9	(Carcinoma, Hepatocellular):ti,ab,kw OR (Carcinomas, Hepatocellular):ti,ab,kw OR (Hepatocellular Carcinomas):ti,ab,kw OR (Liver Cell Carcinoma):ti,ab,kw OR (Carcinoma, Liver Cell):ti,ab,kw (Word variations have been searched)
#10	(Carcinomas, Liver Cell):ti,ab,kw OR (Cell Carcinoma, Liver):ti,ab,kw OR (Cell Carcinomas, Liver):ti,ab,kw OR (Liver Cell Carcinomas):ti,ab,kw OR (Hepatocellular Carcinoma):ti,ab,kw (Word variations have been searched)

#11	(Hepatoma):ti,ab,kw OR (Hepatomas):ti,ab,kw OR (Liver Neoplasms):ti,ab,kw OR (Neoplasms, Hepatic):ti,ab,kw OR (Neoplasms, Liver):ti,ab,kw (Word variations have been searched)
#12	(Liver Neoplasm):ti,ab,kw OR (Neoplasm, Liver):ti,ab,kw OR (Hepatic Neoplasms):ti,ab,kw OR (Hepatic Neoplasm):ti,ab,kw OR (Neoplasm, Hepatic):ti,ab,kw (Word variations have been searched)
#13	(Cancer of Liver):ti,ab,kw OR (Hepatocellular Cancer):ti,ab,kw OR (Cancers, Hepatocellular):ti,ab,kw OR (Hepatocellular Cancers):ti,ab,kw OR (Hepatic Cancer):ti,ab,kw (Word variations have been searched)
#14	(Cancer, Hepatic):ti,ab,kw OR (Cancers, Hepatic):ti,ab,kw OR (Hepatic Cancers):ti,ab,kw OR (Liver Cancer):ti,ab,kw OR (Cancer, Liver):ti,ab,kw (Word variations have been searched)
#15	(Cancer, Liver):ti,ab,kw OR (Cancers, Liver):ti,ab,kw OR (Liver Cancers):ti,ab,kw OR (Cancer of the Liver):ti,ab,kw OR (Cancer, Hepatocellular):ti,ab,kw (Word variations have been searched)
#16	MeSH descriptor: [Liver Neoplasms] explode all trees
#17	#8 or #9 or #10 or #11 or #12 or #13 or #14 or #15 or #16
#18	(TACE):ti,ab,kw OR (transarterial chemoembolization):ti,ab,kw (Word variations have been searched)
#19	MeSH descriptor: [Sorafenib] explode all trees
#20	(Sorafenib):ti,ab,kw OR (Nexavar):ti,ab,kw OR ("BAY 43-9006"):ti,ab,kw OR ("BAY 43 9006"):ti,ab,kw OR ("BAY 439006"):ti,ab,kw (Word variations have been searched)
#21	("BAY-673472"):ti,ab,kw OR ("BAY 673472"):ti,ab,kw OR ("BAY 545-9085"):ti,ab,kw OR ("BAY 545 9085"):ti,ab,kw OR ("BAY 5459085"):ti,ab,kw (Word variations have been searched)
#22	("BAY-545-9085"):ti,ab,kw OR (BAY5459085):ti,ab,kw (Word variations have been searched)

#23	(Lenvatinib):ti,ab,kw OR (Lenvima):ti,ab,kw OR ("E 7080"):ti,ab,kw OR ("E-7080"):ti,ab,kw OR ("ER-203492-00"):ti,ab,kw (Word variations have been searched)
#24	#19 or #20 or #21 or #22
#25	(E7080):ti,ab,kw (Word variations have been searched)
#26	#19 or #20 or #21 or #22
#27	#23 or #26
#28	(regorafenib):ti,ab,kw OR (Stivarga):ti,ab,kw OR ("BAY 73-4506"):ti,ab,kw OR ("BAY73-4506"):ti,ab,kw OR ("BAY-73-4506"):ti,ab,kw (Word variations have been searched)
#29	(apatinib):ti,ab,kw OR (YN968D1):ti,ab,kw OR (YN-968D1):ti,ab,kw OR (rivoceranib):ti,ab,kw (Word variations have been searched)
#30	(anlotinib):ti,ab,kw OR (anlotinib):ti,ab,kw OR (AL3818):ti,ab,kw (Word variations have been searched)
#31	("BMS 907351"):ti,ab,kw OR (BMS907351):ti,ab,kw OR (BMS-907351):ti,ab,kw (Word variations have been searched)
#32	(cabozantinib):ti,ab,kw OR (Cometriq):ti,ab,kw OR ("XL 184"):ti,ab,kw OR ("XL184 cpd"):ti,ab,kw OR ("XL-184"):ti,ab,kw (Word variations have been searched)
#33	#31 or #32
#34	#26 or #27 or #28 or #29 or #30 or #33
#35	#17 and #18 and #34

Search strategy in Webofscience.

# 26	92,614	Title: (Carcinoma, Hepatocellular) OR Title: (Carcinomas, Hepatocellular) OR Title: (Hepatocellular Carcinomas) OR Title: (Liver Cell Carcinoma) OR Title: (Carcinoma, Liver Cell) OR Title: (Carcinomas, Liver Cell) OR Title: (Cell Carcinoma, Liver) OR Title: (Cell Carcinomas, Liver) OR Title: (Liver Cell Carcinomas) OR Title: (Hepatocellular Carcinoma) OR Title: (Hepatoma) OR Title: (Hepatomas) OR Title: (Liver Neoplasms)<br><br>*Index=SCI-EXPANDED, SSCI, A&HCI, CPCI-S, CPCI-SSH, ESCI, CCR-EXPANDED, IC Time=1900-2021*
# 27	16,673	Title: (Neoplasms, Hepatic) OR Title: (Neoplasms, Liver) OR Title: (Liver Neoplasm) OR Title: (Neoplasm, Liver) OR Title: (Hepatic Neoplasms) OR Title: (Hepatic Neoplasm) OR Title: (Neoplasm, Hepatic) OR Title: (Cancer of Liver) OR Title: (Hepatocellular Cancer) OR Title: (Cancers, Hepatocellular) OR Title: (Hepatocellular Cancers) OR Title: (Hepatic Cancer) OR Title: (Cancer, Hepatic)<br><br>Index=SCI-EXPANDED, SSCI, A&HCI, CPCI-S, CPCI-SSH, ESCI, CCR-EXPANDED, IC Time=1900-2021
# 30	20,792	Title: (Cancers, Hepatic) OR Title: (Hepatic Cancers) OR Title: (Liver Cancer) OR Title: (Cancer, Liver) OR Title: (Cancers, Liver) OR Title: (Liver Cancers) OR Title: (Cancer of the Liver) OR Title: (Cancer, Hepatocellular)<br><br>Index=SCI-EXPANDED, SSCI, A&HCI, CPCI-S, CPCI-SSH, ESCI, CCR-EXPANDED, IC Time=1900-2021
# 34	111,886	#30 OR #27 OR #26

		Index=SCI-EXPANDED, SSCI, A&HCI, CPCI-S, CPCI-SSH, ESCI, CCR-EXPANDED, IC Time=1900-2021
# 36	3,675	Title: (TACE) OR Title: (transarterial chemoembolization)<br>*Index=SCI-EXPANDED, SSCI, A&HCI, CPCI-S, CPCI-SSH, ESCI, CCR-EXPANDED, IC Time=1900-2021*
# 40	9,669	Title: (Sorafenib) OR Title: (Nexavar) OR Title: (BAY 43-9006) OR Title: (BAY 43 9006) OR Title: (Lenvatinib) OR Title: (Lenvima) OR Title: (E 7080) OR Title: (E-7080) OR Title: (ER-203492-00) OR Title: (E7080) OR Title: (regorafenib) OR Title: (Stivarga) OR Title: (BAY 73-4506) OR<br>Title: (BAY73-4506) OR Title: (BAY-73-4506)<br>Index=SCI-EXPANDED, SSCI, A&HCI, CPCI-S, CPCI-SSH, ESCI, CCR-EXPANDED, IC Time=1900-2021
# 46	2,169	Title: (apatinib) OR Title: (YN968D1) OR Title: (rivoceranib) OR Title: (anlotinib) OR Title: (AL3818) OR Title: (cabozantinib) OR Title: (Cometriq) OR Title: (XL 184) OR Title: (XL-184) OR Title: (BMS 907351) OR Title: (BMS907351) OR Title: (BMS-907351)<br>Index=SCI-EXPANDED, SSCI, A&HCI, CPCI-S, CPCI-SSH, ESCI, CCR-EXPANDED, IC Time=1900-2021
# 48	11,782	#46 OR #40<br>Index=SCI-EXPANDED, SSCI, A&HCI, ESCI, CCR-EXPANDED, IC Time=1900-2021
# 56	233	#48 AND #36 AND #34<br>Index=SCI-EXPANDED, SSCI Time=All years

Search strategy in Pubmed.

Search number	Query	Results
33	(((("Carcinoma, Hepatocellular"[Mesh]) OR ((((((((((((((((((((((((((((((((((Carcinoma, Hepatocellular[Title/Abstract]) OR (Carcinomas, Hepatocellular[Title/Abstract])) OR (Hepatocellular Carcinomas[Title/Abstract])) OR (Liver Cell Carcinoma[Title/Abstract])) OR (Carcinoma, Liver Cell[Title/Abstract])) OR (Carcinomas, Liver Cell[Title/Abstract])) OR (Cell Carcinoma, Liver[Title/Abstract])) OR (Cell Carcinomas, Liver[Title/Abstract])) OR (Liver Cell Carcinomas[Title/Abstract])) OR (Hepatocellular Carcinoma[Title/Abstract])) OR (Hepatoma[Title/Abstract])) OR (Hepatomas[Title/Abstract])) OR (Liver Neoplasms[Title/Abstract])) OR (Neoplasms, Hepatic[Title/Abstract])) OR (Neoplasms, Liver[Title/Abstract])) OR (Liver Neoplasm[Title/Abstract])) OR (Neoplasm, Liver[Title/Abstract])) OR (Hepatic Neoplasms[Title/Abstract])) OR (Hepatic Neoplasm[Title/Abstract])) OR (Neoplasm, Hepatic[Title/Abstract])) OR (Cancer of Liver[Title/Abstract])) OR (Hepatocellular Cancer[Title/Abstract])) OR (Cancers, Hepatocellular[Title/Abstract])) OR (Hepatocellular Cancers[Title/Abstract])) OR (Hepatic Cancer[Title/Abstract])) OR (Cancer, Hepatic[Title/Abstract])) OR (Cancers, Hepatic[Title/Abstract])) OR (Hepatic Cancers[Title/Abstract])) OR (Liver Cancer[Title/Abstract])) OR (Cancer, Liver[Title/Abstract])) OR (Cancers, Liver[Title/Abstract])) OR (Liver Cancers[Title/Abstract])) OR (Cancer of the Liver[Title/Abstract])) OR (Cancer, Hepatocellular[Title/Abstract]))) OR ("Liver Neoplasms"[Mesh])) AND ((TACE[Title/Abstract]) OR (transarterial chemoembolization[Title/Abstract]))) AND ((((((("Sorafenib"[Mesh]) OR ((((Sorafenib[Title/Abstract]) OR (Nexavar[Title/Abstract])) OR ("BAY 43-9006"[Title/Abstract])) OR ("BAY 43 9006"[Title/Abstract]))) OR (("cabozantinib" [Supplementary Concept]) OR ((((((((cabozantinib[Title/Abstract]) OR (Cometriq[Title/Abstract])) OR ("XL 184"[Title/Abstract])) OR ("XL184 cpd"[Title/Abstract])) OR ("XL-184"[Title/Abstract])) OR ("BMS 907351"[Title/Abstract])) OR ("BMS907351"[Title/Abstract])) OR ("BMS-907351"[Title/Abstract])))) OR (("anlotinib" [Supplementary Concept]) OR ((anlotinib[Title/Abstract]) OR (AL3818[Title/Abstract])))) OR (("apatinib" [Supplementary Concept]) OR ((((apatinib[Title/Abstract]) OR ("YN968D1"[Title/Abstract])) OR ("YN-968D1"[Title/Abstract])) OR (rivoceranib[Title/Abstract])))) OR	710

(("regorafenib" [Supplementary Concept]) OR (((((regorafenib[Title/Abstract]) OR (Stivarga[Title/Abstract])) OR ("BAY 73-4506"[Title/Abstract])) OR ("BAY73-4506"[Title/Abstract])) OR ("BAY-73-4506"[Title/Abstract])))) OR (("lenvatinib" [Supplementary Concept]) OR ((((((Lenvatinib[Title/Abstract]) OR (Lenvima[Title/Abstract])) OR ("E 7080"[Title/Abstract])) OR ("E-7080"[Title/Abstract])) OR ("ER-203492-00"[Title/Abstract])) OR (E7080[Title/Abstract]))))

32	(((((("Sorafenib"[Mesh]) OR ((((Sorafenib[Title/Abstract]) OR (Nexavar[Title/Abstract])) OR ("BAY 43-9006"[Title/Abstract])) OR ("BAY 43 9006"[Title/Abstract]))) OR (("cabozantinib" [Supplementary Concept]) OR ((((((((cabozantinib[Title/Abstract]) OR (Cometriq[Title/Abstract])) OR ("XL 184"[Title/Abstract])) OR ("XL184 cpd"[Title/Abstract])) OR ("XL-184"[Title/Abstract])) OR ("BMS 907351"[Title/Abstract])) OR ("BMS907351"[Title/Abstract])) OR ("BMS-907351"[Title/Abstract])))) OR (("anlotinib" [Supplementary Concept]) OR ((anlotinib[Title/Abstract]) OR (AL3818[Title/Abstract])))) OR (("apatinib" [Supplementary Concept]) OR ((((apatinib[Title/Abstract]) OR ("YN968D1"[Title/Abstract])) OR ("YN-968D1"[Title/Abstract])) OR (rivoceranib[Title/Abstract])))) OR (("regorafenib" [Supplementary Concept]) OR (((((regorafenib[Title/Abstract]) OR (Stivarga[Title/Abstract])) OR ("BAY 73-4506"[Title/Abstract])) OR ("BAY73-4506"[Title/Abstract])) OR ("BAY-73-4506"[Title/Abstract])))) OR (("lenvatinib" [Supplementary Concept]) OR ((((((Lenvatinib[Title/Abstract]) OR (Lenvima[Title/Abstract])) OR ("E 7080"[Title/Abstract])) OR ("E-7080"[Title/Abstract])) OR ("ER-203492-00"[Title/Abstract])) OR (E7080[Title/Abstract])))	13,473
31	("Sorafenib"[Mesh]) OR ((((Sorafenib[Title/Abstract]) OR (Nexavar[Title/Abstract])) OR ("BAY 43-9006"[Title/Abstract])) OR ("BAY 43 9006"[Title/Abstract]))	10,006
30	(((Sorafenib[Title/Abstract]) OR (Nexavar[Title/Abstract])) OR ("BAY 43-9006"[Title/Abstract])) OR ("BAY 43 9006"[Title/Abstract])	9,401
8	"Sorafenib"[Mesh]	5,379
29	("cabozantinib" [Supplementary Concept]) OR ((((((((cabozantinib[Title/Abstract]) OR (Cometriq[Title/Abstract])) OR ("XL 184"[Title/Abstract])) OR ("XL184 cpd"[Title/Abstract])) OR ("XL-184"[Title/Abstract])) OR ("BMS 907351"[Title/Abstract])) OR ("BMS907351"[Title/Abstract])) OR ("BMS-907351"[Title/Abstract]))	1,131
28	(((((((cabozantinib[Title/Abstract]) OR (Cometriq[Title/Abstract])) OR ("XL 184"[Title/Abstract])) OR ("XL184 cpd"[Title/Abstract])) OR ("XL-184"[Title/Abstract])) OR ("BMS 907351"[Title/Abstract])) OR ("BMS907351"[Title/Abstract])) OR ("BMS-907351"[Title/Abstract])	1,081
27	"cabozantinib" [Supplementary Concept]	453
26	("anlotinib" [Supplementary Concept]) OR ((anlotinib[Title/Abstract]) OR (AL3818[Title/Abstract]))	277

25	(anlotinib[Title/Abstract]) OR (AL3818[Title/Abstract])	275
24	"anlotinib" [Supplementary Concept]	102
23	("apatinib" [Supplementary Concept]) OR ((((apatinib[Title/Abstract]) OR ("YN968D1"[Title/Abstract])) OR ("YN-968D1"[Title/Abstract])) OR (rivoceranib[Title/Abstract]))	765
22	(((apatinib[Title/Abstract]) OR ("YN968D1"[Title/Abstract])) OR ("YN-968D1"[Title/Abstract])) OR (rivoceranib[Title/Abstract])	762
21	"apatinib" [Supplementary Concept]	385
20	((((((((((Sorafenib[Title/Abstract]) OR (Nexavar[Title/Abstract])) OR ("BAY 43-9006"[Title/Abstract])) OR ("BAY 43 9006"[Title/Abstract])) OR ("BAY 439006"[Title/Abstract])) OR ("BAY-673472"[Title/Abstract])) OR ("BAY 673472"[Title/Abstract])) OR ("BAY 545-9085"[Title/Abstract])) OR ("BAY 545 9085"[Title/Abstract])) OR ("BAY 5459085"[Title/Abstract])) OR ("BAY-545-9085"[Title/Abstract])) OR ("BAY5459085"[Title/Abstract])	9,401
19	("regorafenib" [Supplementary Concept]) OR (((((regorafenib[Title/Abstract]) OR (Stivarga[Title/Abstract])) OR ("BAY 73-4506"[Title/Abstract])) OR ("BAY73-4506"[Title/Abstract])) OR ("BAY-73-4506"[Title/Abstract]))	1,458
18	((((regorafenib[Title/Abstract]) OR (Stivarga[Title/Abstract])) OR ("BAY 73-4506"[Title/Abstract])) OR ("BAY73-4506"[Title/Abstract])) OR ("BAY-73-4506"[Title/Abstract])	1,404
17	"regorafenib" [Supplementary Concept]	653
16	("lenvatinib" [Supplementary Concept]) OR ((((((Lenvatinib[Title/Abstract]) OR (Lenvima[Title/Abstract])) OR ("E 7080"[Title/Abstract])) OR ("E-7080"[Title/Abstract])) OR ("ER-203492-00"[Title/Abstract])) OR (E7080[Title/Abstract]))	1,172
15	(((((Lenvatinib[Title/Abstract]) OR (Lenvima[Title/Abstract])) OR ("E 7080"[Title/Abstract])) OR ("E-7080"[Title/Abstract])) OR ("ER-203492-00"[Title/Abstract])) OR (E7080[Title/Abstract])	1,136
14	"lenvatinib" [Supplementary Concept]	442

13	("Sorafenib"[Mesh]) OR ((((((((((((Sorafenib[Title/Abstract])) OR (Nexavar[Title/Abstract])) OR ("BAY 43-9006"[Title/Abstract])) OR ("BAY 43 9006"[Title/Abstract])) OR ("BAY 439006"[Title/Abstract])) OR ("BAY-673472"[Title/Abstract])) OR ("BAY 673472"[Title/Abstract])) OR ("BAY 545-9085"[Title/Abstract])) OR ("BAY 545 9085"[Title/Abstract])) OR ("BAY 5459085"[Title/Abstract])) OR ("BAY-545-9085"[Title/Abstract])) OR ("BAY5459085"[Title/Abstract]))	10,006
12	"BAY 5459085"[Title/Abstract]	0
11	"BAY 5459085"[Title/Abstract] - Schema: all	0
10	(((((((((((Sorafenib[Title/Abstract])) OR (Nexavar[Title/Abstract])) OR ("BAY 43-9006"[Title/Abstract])) OR ("BAY 43 9006"[Title/Abstract])) OR ("BAY 439006"[Title/Abstract])) OR ("BAY-673472"[Title/Abstract])) OR ("BAY 673472"[Title/Abstract])) OR ("BAY 545-9085"[Title/Abstract])) OR ("BAY 545 9085"[Title/Abstract])) OR ("BAY 5459085"[Title/Abstract])) OR ("BAY-545-9085"[Title/Abstract])) OR ("BAY5459085"[Title/Abstract])	9,401
9	((((((((((Sorafenib[Title/Abstract]) OR (Nexavar[Title/Abstract])) OR (BAY 43-9006[Title/Abstract])) OR (BAY 43 9006[Title/Abstract])) OR (BAY 439006[Title/Abstract])) OR (BAY-673472[Title/Abstract])) OR (BAY 673472[Title/Abstract])) OR (BAY 545-9085[Title/Abstract])) OR (BAY 545 9085[Title/Abstract])) OR (BAY 5459085[Title/Abstract])) OR (BAY-545-9085[Title/Abstract])) OR (BAY5459085[Title/Abstract])	9,401
7	Sorafenib[MeSH Terms]	5,379
6	(TACE[Title/Abstract]) OR (transarterial chemoembolization[Title/Abstract])	7,489
5	TACE	6,127

4	(("Carcinoma, Hepatocellular"[Mesh]) OR ((((((((((((((((((((((((((((((((((Carcinoma, Hepatocellular[Title/Abstract]) OR (Carcinomas, Hepatocellular[Title/Abstract])) OR (Hepatocellular Carcinomas[Title/Abstract])) OR (Liver Cell Carcinoma[Title/Abstract])) OR (Carcinoma, Liver Cell[Title/Abstract])) OR (Carcinomas, Liver Cell[Title/Abstract])) OR (Cell Carcinoma, Liver[Title/Abstract])) OR (Cell Carcinomas, Liver[Title/Abstract])) OR (Liver Cell Carcinomas[Title/Abstract])) OR (Hepatocellular Carcinoma[Title/Abstract])) OR (Hepatoma[Title/Abstract])) OR (Hepatomas[Title/Abstract])) OR (Liver Neoplasms[Title/Abstract])) OR (Neoplasms, Hepatic[Title/Abstract])) OR (Neoplasms, Liver[Title/Abstract])) OR (Liver Neoplasm[Title/Abstract])) OR (Neoplasm, Liver[Title/Abstract])) OR (Hepatic Neoplasms[Title/Abstract])) OR (Hepatic Neoplasm[Title/Abstract])) OR (Neoplasm, Hepatic[Title/Abstract])) OR (Cancer of Liver[Title/Abstract])) OR (Hepatocellular Cancer[Title/Abstract])) OR (Cancers, Hepatocellular[Title/Abstract])) OR (Hepatocellular Cancers[Title/Abstract])) OR (Hepatic Cancer[Title/Abstract])) OR (Cancer, Hepatic[Title/Abstract])) OR (Cancers, Hepatic[Title/Abstract])) OR (Hepatic Cancers[Title/Abstract])) OR (Liver Cancer[Title/Abstract])) OR (Cancer, Liver[Title/Abstract])) OR (Cancers, Liver[Title/Abstract])) OR (Liver Cancers[Title/Abstract])) OR (Cancer of the Liver[Title/Abstract])) OR (Cancer, Hepatocellular[Title/Abstract]))) OR ("Liver Neoplasms"[Mesh])	227,077
3	"Liver Neoplasms"[Mesh]	175,467

2	(((((((((((((((((((((((((((((((((Carcinoma, Hepatocellular[Title/Abstract]) OR (Carcinomas, Hepatocellular[Title/Abstract])) OR (Hepatocellular Carcinomas[Title/Abstract])) OR (Liver Cell Carcinoma[Title/Abstract])) OR (Carcinoma, Liver Cell[Title/Abstract])) OR (Carcinomas, Liver Cell[Title/Abstract])) OR (Cell Carcinoma, Liver[Title/Abstract])) OR (Cell Carcinomas, Liver[Title/Abstract])) OR (Liver Cell Carcinomas[Title/Abstract])) OR (Hepatocellular Carcinoma[Title/Abstract])) OR (Hepatoma[Title/Abstract])) OR (Hepatomas[Title/Abstract])) OR (Liver Neoplasms[Title/Abstract])) OR (Neoplasms, Hepatic[Title/Abstract])) OR (Neoplasms, Liver[Title/Abstract])) OR (Liver Neoplasm[Title/Abstract])) OR (Neoplasm, Liver[Title/Abstract])) OR (Hepatic Neoplasms[Title/Abstract])) OR (Hepatic Neoplasm[Title/Abstract])) OR (Neoplasm, Hepatic[Title/Abstract])) OR (Cancer of Liver[Title/Abstract])) OR (Hepatocellular Cancer[Title/Abstract])) OR (Cancers, Hepatocellular[Title/Abstract])) OR (Hepatocellular Cancers[Title/Abstract])) OR (Hepatic Cancer[Title/Abstract])) OR (Cancer, Hepatic[Title/Abstract])) OR (Cancers, Hepatic[Title/Abstract])) OR (Hepatic Cancers[Title/Abstract])) OR (Liver Cancer[Title/Abstract])) OR (Cancer, Liver[Title/Abstract])) OR (Cancers, Liver[Title/Abstract])) OR (Liver Cancers[Title/Abstract])) OR (Cancer of the Liver[Title/Abstract])) OR (Cancer, Hepatocellular[Title/Abstract])	149,278
1	"Carcinoma, Hepatocellular"[Mesh]	91,765

Supplementary Material S2. PRISMA flow diagram.

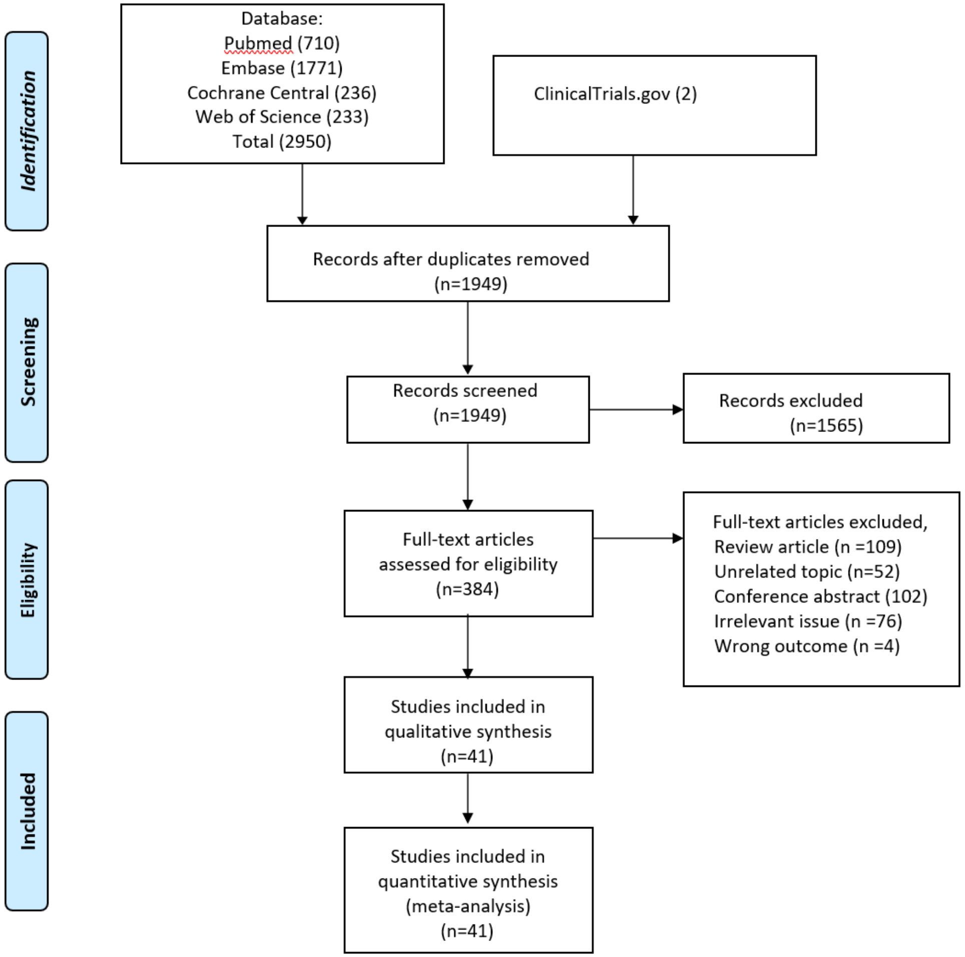

Supplementary Material S3. Characteristics of included studies in this analysis.

	author	year	Enrollment period	center	country	Group	number	Gender (M/F)	Age	BCLC (A/B/C)	tumor size	Tumor number (Single/Multiple)	Vascular invasion (Yes/No)	EHS (Yes/No)
1	Zou et al.	2021	Jun. 2012 to Nov. 2017	1	China	TACEplusSorafenib	42	32/10	58.31±7.83	0/23/19	＞5:27<br>3-5:12<br>⩽:3:3	＞3:30<br>⩽3:12	/	4/38
						TACE	43	31/12	58.53±8.11	0/25/18	＞5:26<br>3-5:13<br>⩽:3:4	＞3:29<br>⩽3:14	/	3/40
2	Qiu et al.	2021	Jan. 2016 to Jun. 2018	3	China	TACEplusSorafenib	115	104/11	＜52:53<br>⩾52:62	/	＞5:81<br>⩽5:34	35/80	71/44	66/49
						TACEplusApatinib	86	78/8	＜52:42<br>⩾52:44	/	＞5:53<br>⩽5:33	32/54	55/31	50/36
3	Kuang et al.	2021	May. 2016 to Oct. 2017	1	China	TACEplusSorafenib	66	49/17	60.46±9.91	0/29/37	5.23±2.49	16/50	17/49	11/55
						TACE	66	55/11	58.97±10.52	0/24/42	5.41±2.31	23/43	21/45	8/58
4	Koch et al.	2021	Jan. 2007 to Dec. 2012	3	Germany	TACEplusSorafenib	54	47/7	64(34-77)	0/54	/	/	18/36	22/32
						TACE	65	57/9	67(41-80)	0/65	/	/	25/40	22/43
5	Fu et al.	2021	Jul. 2017 to Oct. 2019	1	China	TACEplusLenvatinib	60	50/10	60 (25–76)	2/33/25	＞3:51<br>⩽3:9	9/51	21/39	9/51
						TACE	60	55/5	60 (33–81)	3/26/31	＞3:54<br>⩽3:6	10/50	27/33	9/51
6	Ding et al.	2021	Dec. 2018 to May. 2020	1	China	TACEplusLenvatinib	32	25/7	57 ± 11	/	10.8 ± 4.2	16/16	/	13/19
						TACEplusSorafenib	32	27/5	56 ± 11	/	10.1 ± 3.9	13/19	/	9/23

7	Cao et al.	2021	Jun. 2015 to Mar. 2020	1	China	TACEplusSorafenib	32	28/4	52.7 ± 12.4	/	＞5:5 ⩽5:27	＞3:8 ⩽3:24	/	18/14
						TACEplusApatinib	41	37/4	51.6 ± 9.6	/	＞5:14 ⩽5:27	＞3:5 ⩽3:36	/	24/17
8	Ando et al.	2021	Mar. 2018 to Aug. 2020	2	Japan	TACEplusLenvatinib	19	18/1	74 (46–87)	/	30 (10–100)	9/21	/	/
						TACE	19	17/2	72 (57–88)	/	23(10–120)	20/38	/	/
9	Wang et al.	2020	Jan. 2009 to Dec. 2015	17	China	TACE	1,406	1183/223	56.7±12.1	192/756/458	7.7±4.0	1 (1–2)	/	/
						TACEplusSorafenib	313	267/46	53.7±12.0	36/167/110	7.9±4.0	2 (1–3)	/	/
10	Shen et al.	2020	Jan. 2013 to Jun. 2019	1	China	TACE	280	265/15	＜52:134 ⩾52:146	/	＞5:252 ⩽5:28	130/150	/	62/218
						TACEplusApatinib	40	38/2	＜52:17 ⩾52:23	/	＞5:31 ⩽5:9	14/26	/	13/27
11	liu et al.	2020	Jun. 2014 to Jun. 2016	1	China	TACEplusSorafenib	59	37/22	56.31±9.87	0/30/29	＞5:33 ⩽5:26	38/21	/	/
						TACE	59	32/27	58.11±10.44	0/36/23	＞5:37 ⩽5:22	41/18	/	/
12	liu et al.	2020	Jan. 2010 to Dec. 2018	1	China	TACEplusSorafenib	35	30/5	68.24 (35–88)	/	6.9 (1.6–12)	18/17	24/11	11/24
						TACE	40	32/8	56 (35–85)	/	7.4 (2.1–11.7)	27/13	28/12	18/22
13	liu et al.	2020	Jul. 2016 to Dec. 2017	1	China	TACEplusApatinib	34	29/5	53.3 ± 9.4	0/18/16	11.7 ± 2.1	/	16/18	5/29
						TACE	48	39/9	56.5 ± 9.7	0/28/20	11.8 ± 2.2	/	21/27	8/40

14	lee et al.	2020	Aug. 2012 to Sep. 2017	1	China	Sorafenib	65	54/	64.28 ± 12.66	/	＞5:31<br>3-5:17<br>⩽:3:17	＞3:43<br>⩽3:22	/	35/30
						TACEplusSorafenib	53	46/	61.74 ± 12.25	/	＞5:15<br>3-5:20<br>⩽:3:18	＞3:34<br>⩽3:19	/	26/27
15	Kimura et al.	2020	Apr. 2009 to Jun. 2018	1	Japan	Sorafenib	32	27/5	73.5 (65.8 - 78.1)	0/9/23	/	/	/	11/21
						TACEplusSorafenib	14	10/4	71.8 (69.6 - 78.8)	0/10/4	/	/	/	9/5
16	Kan et al.	2020	Jun. 2015 to Sep. 2018	1	China	TACEplusApatinib	126	112/14	50.5 ± 10.3	/	/	/	70/56	62/64
						TACE	97	81/16	53.7 ± 10.3	/	/	/	62/35	50/47
17	Guo et al.	2020	Jan. 2018 to Jan. 2019	1	China	TACEplusAnlotinib	36	29/7	56.41 + 12.44	3/16/17	90.37 + 46.89	14/22	12/24	8/28
						TACE	46	38/8	56.28 + 11.66	1/23/22	90.44 + 31.97	14/32	20/26	14/32
18	Gu et al.	2020	Jan. 2018 to Jan. 2020	1	China	TACE	40	22/18	51 (33–67)	0/33/7	＞5:11<br>⩽5:29	17/23	/	/
						TACEplusApatinib	40	19/21	53 (31–68)	0/36/4	＞5:9<br>⩽5:31	21/19	/	/
19	Cao et al	2020	Dec.2011 to Dec 2017	1	China	TACEplusSorafenib	50	/		/	/	/	/	/
						TACE	50	/		/	/	/	/	/
20	Yuan et al.	2019	Jan. 2008, to Apr. 2014	1	China	TACEplusSorafenib	69	59/10	51 (21-79)	/	8.39±4.45	＞3:34<br>⩽3:19	69/0	/
						TACE	429	380/49	51 (18-84)	/	9.65±3.25	＞3:34<br>⩽3:19	429/0	/

21	Yang et al.	2019	Sep. 2016 to Aug. 2017	1	China	TACEplusApatinib	25	20/5	＜65:13 ⩾65:12	0/10/15	/	/	/	/
						TACE	22	18/4	＜65:12 ⩾65:10	0/10/12	/	/	/	/
22	Ren et al.	2019	Feb. 2008 to Aug. 2015	1	China	TACEplusSorafenib	61	48/13		0/30/31	＞5:35 ⩽5:26	40/21	20/41	14/47
						TACE	247	209/38		0/150/97	＞5:190 ⩽5:57	127/120	71/176	15/232
23	Park et al.	2019	Jan. 2013 to Dec. 2015	12	Korea	TACEplusSorafenib	170	136/	60.2 (9.6)	3/39/128	/	/	68/102	62/108
						Sorafenib	169	147/	61.3 (9.6)	0/44/125	/	/	63/106	59/110
24	Kudo et al.	2019	Feb. 2011 to Mar. 2016	33	Japan	TACEplusSorafenib	80	63/17	72.0 (36–85)	27/44/9	/	/	/	/
						TACE	76	55/21	73.0 (53–86)	33/34/9	/	/	/	/
25	Kok et al.	2019	Aug. 2012 to Dec. 2013	/	Japan	TACEplusSorafenib	426	355/71	60.4(50.7-68.7)	/	/	/	/	197/229
						SOR	3248	2528/720	63.1(55.2-72.5)	/	/	/	/	1255/1993
26	Fan et al.	2019	Jan. 2015 to Jan. 2016	4	China	TACEplusApatinib	85	68/17	49 (17-71)	/	＞7:58 ⩽7:27	17/68	85/0	/
						TACE	103	71/32	50 (19-80)	/	＞7:80 ⩽7:23	26/77	103/0	/
27	Zhu et al	2018	Jan. 2010 to Dec. 2012	1	China	TACEplusSorafenib	46	39/7	48.4 6 8.1	/	/	/	46/0	17/29
						TACE	45	38/7	51.9 6 12.2	/	/	/	45/0	18/27

28	Lei et al	2018	Jan. 2009 to Dec. 2011	1	China	TACEplusSorafenib	38	24/14	52 ± 5	/	＞7:14<br>⩽7:24	/	/	/
						TACE	29	18/11	51 ± 6	/	＞7:10<br>⩽7:19	/	/	/
29	Yao et al	2017	Oct. 2009 to Feb. 2015	1	China	TACE	78	78/0	46.67±8.5	18/60/	6.23±3.86	/	/	/
						TACEplusSorafenib	19	19/0	45.32±5.94	5/14/	5.67±3.54	/	/	/
30	Wu et al	2017	Aug. 2004 to Nov. 2014	1	China	Sorafenib	56	48/8	50.23 ± 11.88	0/10/46	9.1 (1,19.5)	2 (1,4)	37/19	17/39
						TACEplusSorafenib	48	46/2	47.6 ± 12.73	0/16/32	7.65 (1,19)	2 (1,4)	26/22	21/27
31	Meyer et al	2017	Nov. 2010 to Dec. 2015	20	UK	TACEplusSorafenib	57	139/18	65 (57–71)	/	6 (4–8)	/	/	/
						TACE	156	138/18	68 (63–74)	/	5 (4–8)	/	/	/
32	Huang et al	2017	Jan. 2008 to Jan. 2014	1	China	TACEplusSorafenib	46	43/3	50.2 ± 11.9	0/46/	/	13/33	/	/
						TACE	144	133/11	55.4 ± 14.1	0/144/	/	43/101	/	/
33	Zhang et al	2016	Feb. 2009 to Oct. 2013	1	China	TACEplusSorafenib	20	18/2	46.6±11.0	/	9.6±4.0	6/30/14	8/12	4/16
						TACE	60	58/2	48.6±7.1	/	10.3±3.4	22/37/38	29/31	12/48
34	Wan et al	2016	Jul. 2007 to Dec. 2011	1	China	TACEplusSorafenib	245	218/27	＜50:115<br>⩾50:130	/	＞5:92<br>⩽5:153	107/138	70/175	43/202
						TACE	245	218/27	＜50:115<br>⩾50:130	/	＞5:92<br>⩽5:153	107/138	27/218	36/209
35	Zhang et al	2015	Jan. 2009 to Jun. 2013	1	China	TACEplusSorafenib	45	43/2	50.1 ± 8.8	/	/	/	/	/
						Sorafenib	44	41/3	53.6 ± 9.7	/	/	/	/	/

36	Lu et al	2017	Mar. 2015 to Aug. 2015	1	China	TACE	22	17/5	58.9 ± 9.38	0/19/3	6.86 ± 2.12	/	46/0	17/29
						TACEplusApatinib	20	16/4	56.1 ± 10.79	0/18/2	7.12 ± 2.15	/	45/0	18/27
37	Hu et al	2014	Feb. 2009 to Nov. 2011	1	China	TACEplusSorafenib	82	69/13	61 ± 11	/	/	39/43	/	27/55
						TACE	198	159/39	57 ± 12	/	/	90/108	/	60/138
38	Muhammad et al	2013	Jan. 2007 to Dec. 2011	1	USA	TACEplusSorafenib	13	/	61.4 ± 7.5	6/2/5	4 (1.5 - 16.7)	/	/	/
						TACE	30	/	59.2 ± 7.4	22/8/0	3.1 (1.4 - 5.8)	/	/	/
39	Choi et al	2013	Apr. 2007 to Jul. 2011	1	Korea	TACEplusSorafenib	164	139/25	52 (26–75)	/	/	6/158	106/58	128/36
						Sorafenib	191	166/25	54 (22–84)	/	/	9/182	94/97	155/36
40	Bai et al	2013	Mar. 2008 to Nov. 2009	1	China	TACEplusSorafenib	82	73/9	54 ± 13	0/19/63	/	/	11/34	24/34
						TACE	222	188/34	50 ± 12	0/81/141	/	/	20/120	58/120
41	Qu et al	2012	Jun. 2008 to Feb. 2011	1	China	TACEplusSorafenib	45	41/4	51 ± 11.7	0/16/29	＞5:35 ⩽5:10	/	20/25	11/34
						TACE	45	41/4	49 ± 11.0	0/17/28	＞5:36 ⩽5:9	/	23/22	12/33

Supplementary Material S4. Results of heterogeneity analysis

The “mtc.anohe” command in the “gemtc” package was used to evaluate global heterogeneity, which was documented using the variance parameter I2. Further, a value >50% was considered indicative of considerable heterogeneity.

	MIC		I^2		MIC		I^2	
	consistency model (random)	inconsistency model (random)	consistency model (random)	inconsistency model (random)	consistency model (fixed)	inconsistency model (fixed)	consistency model (fixed)	inconsistency model (fixed)
**OS**	75.84542	75.96552	25%	25%	123.153273	115.916687	69%	66%
**PFS**	27.46072	27.30269	3%	5%	39.567567	29.223761	57%	33%
**CR**	72.12656	72.72673	0%	0%	87.59672	71.71653	15%	0%
**PR**	110.07907	110.07624	6%	6%	152.36579	142.51188	52%	46%
**SD**	105.66935	107.00333	8%	8%	109.7584	110.36188	25%	23%
**PD**	100.60118	102.18396	0%	2%	114.35746	107.58152	30%	21%
**ORR**	111.78902	111.8657	8%	8%	164.10899	150.9305	56%	50%
**DCR**	110.98869	111.92789	9%	10%	189.60323	159.2091	64%	55%

Supplementary Material S5. Quality assessment of the included studies

Newcastle-Ottawa Scale for assessing the quality of cohort studies (9-point)									
**Author**	**Representativeness of the exposed cohort**	**Selection of the nonexposed cohort**	**Ascertainment of exposure**	**Demonstration that outcome of interest was not present at start of study**	**Comparability of cohorts on the basis of the design or analysis**	**Assessment of outcome**	**Was follow-up long enough for outcomes to occur**	**Adequacy of follow up of cohorts**	**Total scores**
Zou et al.2021	1	1	1	1	1	1	1	1	8
Qiu et al.2021	1	1	1	1	2	1	1	1	9
Kuang et al.2021	1	1	1	1	1	1	1	1	8
Koch et al.2021	1	1	1	1	1	1	1	1	8
Fu et al.2021	1	1	1	1	1	1	1	1	8
Cao et al.2021	1	1	1	1	2	1	1	1	9
Ando et al.2021	1	1	1	1	2	1	1	1	9
Wang et al.2020	1	1	1	1	2	1	1	1	9
Shen et al.2020	1	1	1	1	2	1	1	1	9
liu et al.2020	1	1	1	1	1	1	1	1	8
liu et al.2020	1	1	1	1	1	1	1	1	8
liu et al.2020	1	1	1	1	2	1	1	1	9
lee et al.2020	1	1	1	1	2	1	1	1	9
Kimura et al.2020	1	1	1	1	1	1	1	1	8
Kan et al.2020	1	1	1	1	2	1	1	1	9

Guo et al.2020	1	1	1	1	2	1	1	1	9
Cao et al.2020	1	1	1	1	1	1	1	1	8
Yuan et al.2019	1	1	1	1	1	1	1	1	8
Yang et al.2019	1	1	1	1	1	1	1	1	8
Ren et al.2019	1	1	1	1	1	1	1	1	8
Kok et al.2019	1	1	1	1	1	1	1	1	8
Fan et al.2019	1	1	1	1	2	1	1	1	9
Zhu et al.2018	1	1	1	1	2	1	1	1	9
Lei et al.2018	1	1	1	1	1	1	1	1	8
Yao et al.2017	1	1	1	1	1	1	1	1	8
Wu et al.2017	1	1	1	1	1	1	1	1	8
Huang et al.2017	1	1	1	1	1	1	1	1	8
Zhang et al.2016	1	1	1	1	1	1	1	1	8
Wan et al.2016	1	1	1	1	1	1	1	1	8
Zhang et al.2015	1	1	1	1	1	1	1	1	8
Zhu et al.2014	1	1	1	1	2	1	1	1	9
Hu et al.2014	1	1	1	1	2	1	1	1	9
Muhammad et al.2013	1	1	1	1	1	1	1	1	8
Choi et al.2013	1	1	1	1	2	1	1	1	9
Bai et al.2013	1	1	1	1	2	1	1	1	9
Qu et al.2012	1	1	1	1	2	1	1	1	9

Methodology Quality Accessment-Modified Jadad Score (7-point)					
Author	Randomization	Concealment of allocation	Double blinding	Withdrawls and dropouts	Total scores
Ding et al.2021	2	2	0	1	5
Gu et al.2020	2	2	0	0	4
Park et al.2019	2	2	0	1	5
Kudo et al.2019	2	2	0	1	5
Meyer et al.2017	2	2	2	1	7

Supplementary Material S6. Results of convergence

Supplementary Material S6.1 Brooks-Gelman-Rubin plot

OS

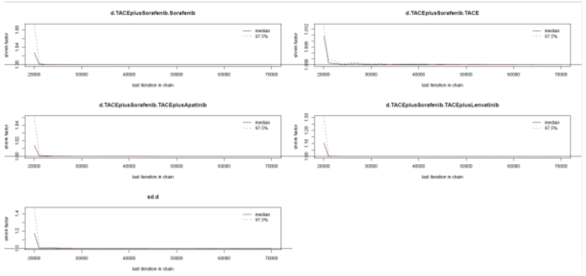

PFS

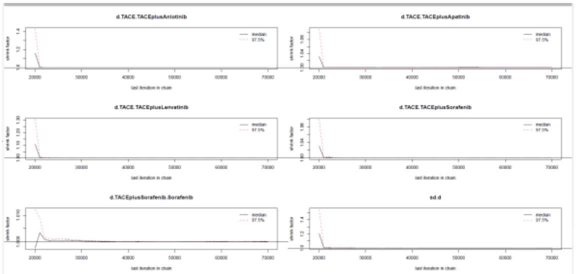

CR

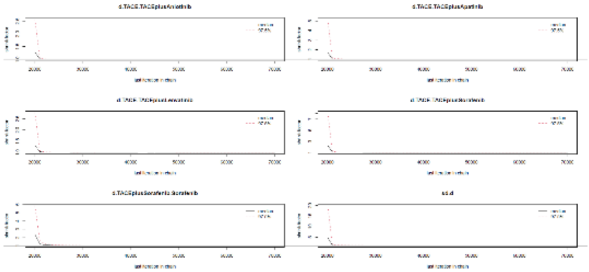

PR

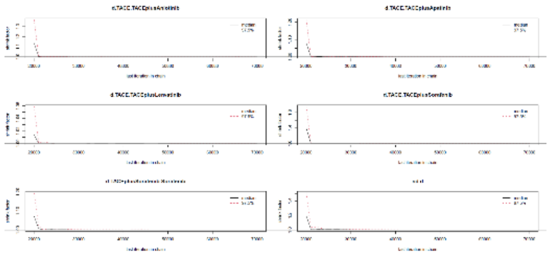

SD

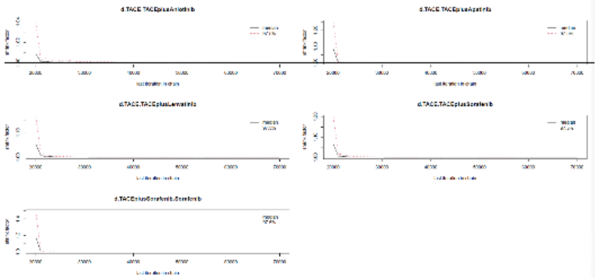

PD

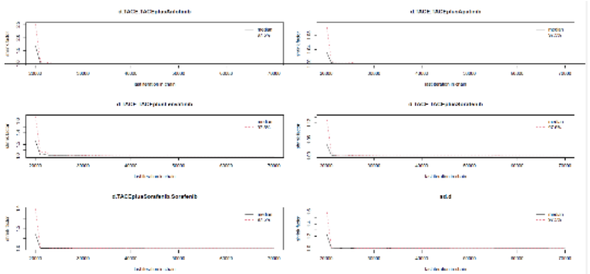

ORR

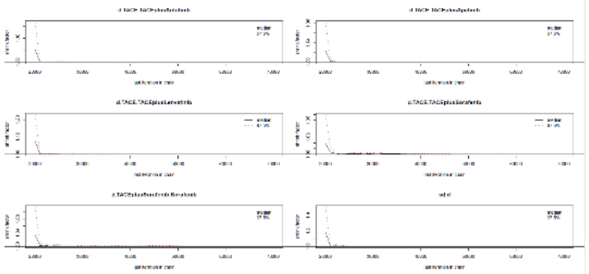

DCR

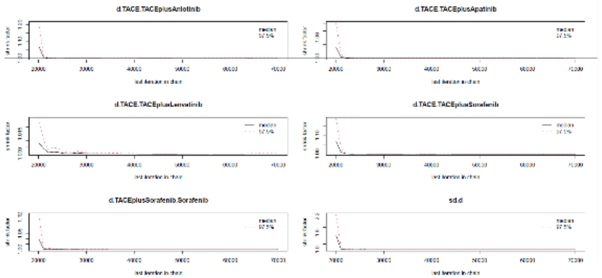

Supplementary Material S6.2 Trace plot and density plot

OS

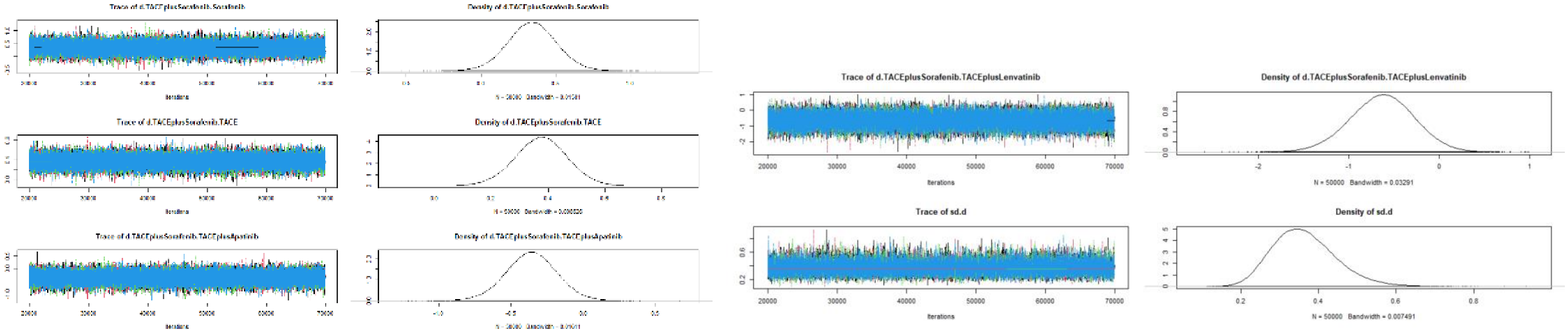

PFS

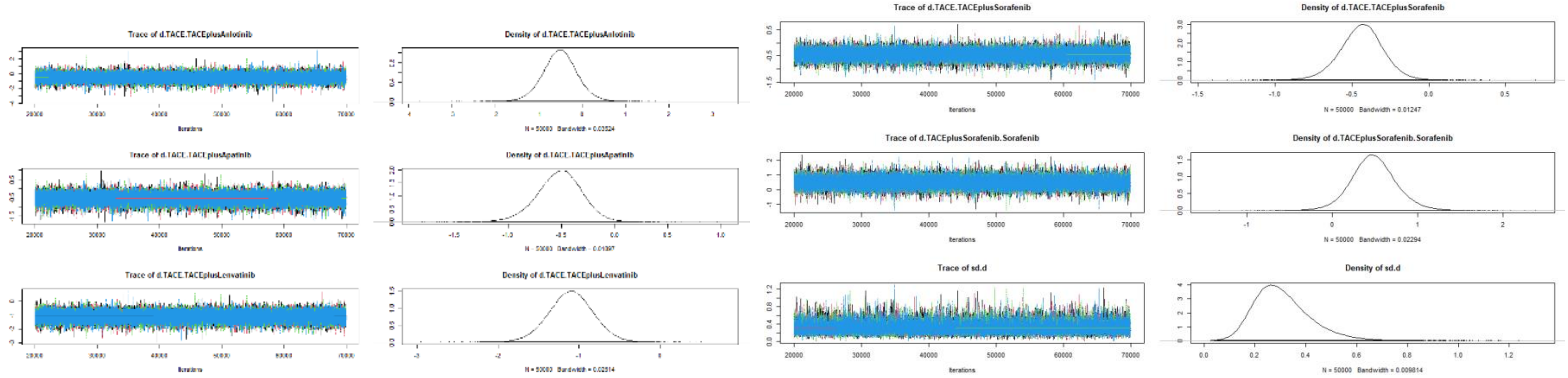

CR

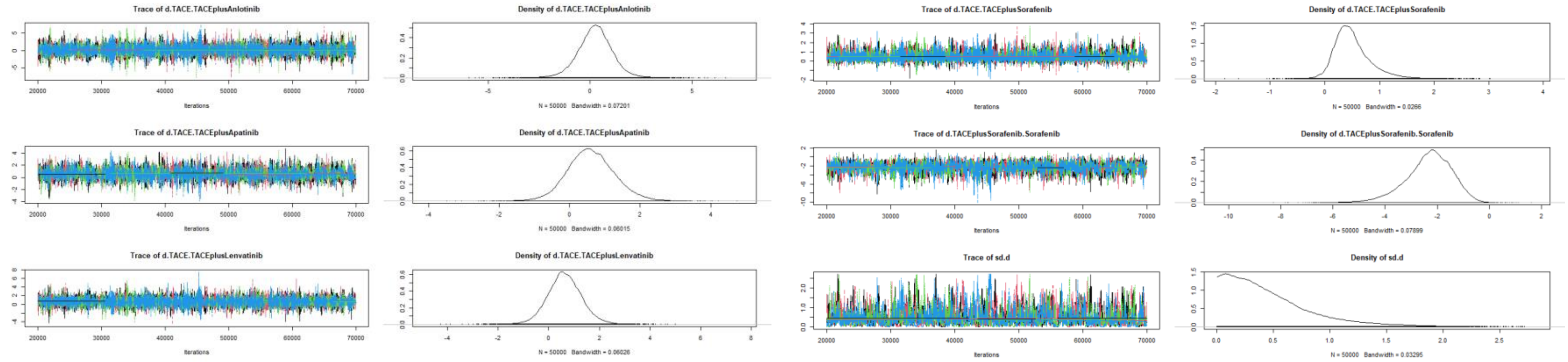
Trace of d.TACE.TACEplusAnlotinib
Density of d.TACE.TACEplusAnlotinib
N = 50000 Bandwidth = 0.07201
Trace of d.TACE.TACEplusSorafenib
Density of d.TACE.TACEplusSorafenib
N = 50000 Bandwidth = 0.0266
Trace of d.TACE.TACEplusApatinib
Density of d.TACE.TACEplusApatinib
N = 50000 Bandwidth = 0.06015
Trace of d.TACEplusSorafenib.Sorafenib
Density of d.TACEplusSorafenib.Sorafenib
N = 50000 Bandwidth = 0.07899
Trace of d.TACE.TACEplusLenvatinib
Density of d.TACE.TACEplusLenvatinib
N = 50000 Bandwidth = 0.06026
Trace of sd.d
Density of sd.d
N = 50000 Bandwidth = 0.03295
Iterations

PR

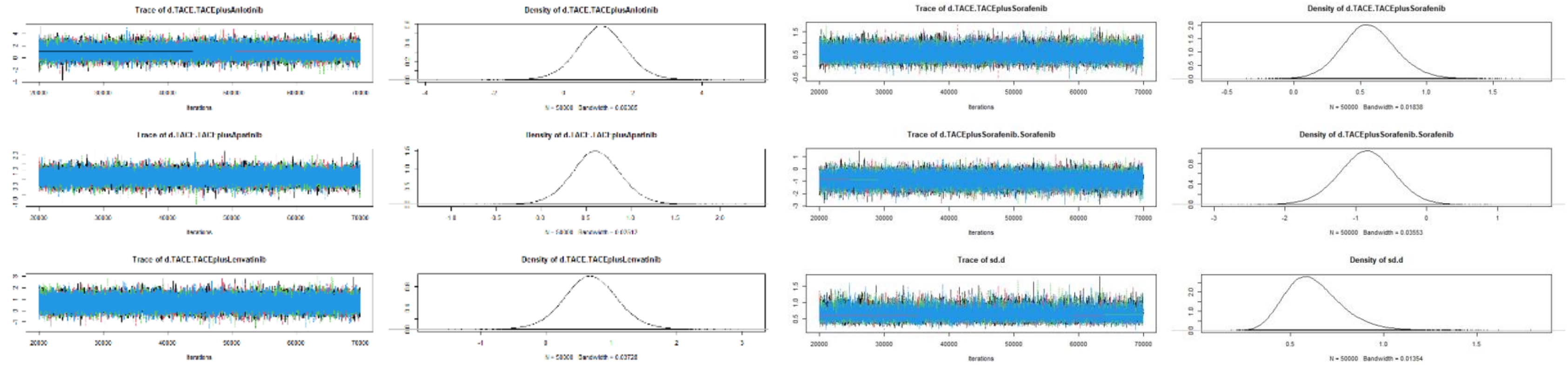
Trace of d.TACE.TACEplusAnlotinib
Density of d.TACE.TACEplusAnlotinib
Trace of d.TACE.TACEplusSorafenib
Density of d.TACE.TACEplusSorafenib
N = 50000 Bandwidth = 0.01838
Trace of d.TACEplusSorafenib.Sorafenib
Density of d.TACEplusSorafenib.Sorafenib
N = 50000 Bandwidth = 0.03553
Trace of d.TACE.TACEplusLenvatinib
Density of d.TACE.TACEplusLenvatinib
Trace of sd.d
Density of sd.d
N = 50000 Bandwidth = 0.01354
Iterations

SD

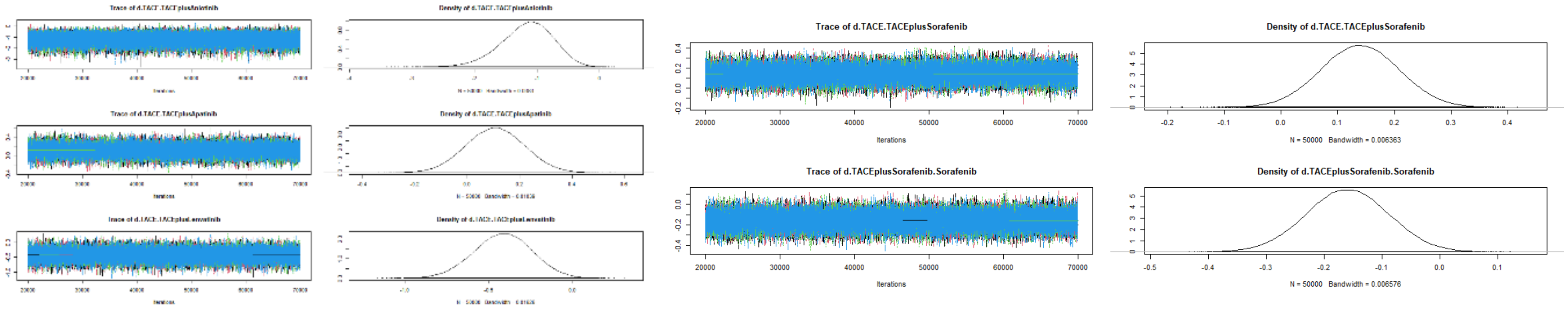
Trace of d.TACE.TACEplusSorafenib
Iterations
Density of d.TACE.TACEplusSorafenib
N = 50000 Bandwidth = 0.006363
Trace of d.TACEplusSorafenib.Sorafenib
Iterations
Density of d.TACEplusSorafenib.Sorafenib
N = 50000 Bandwidth = 0.006576

PD

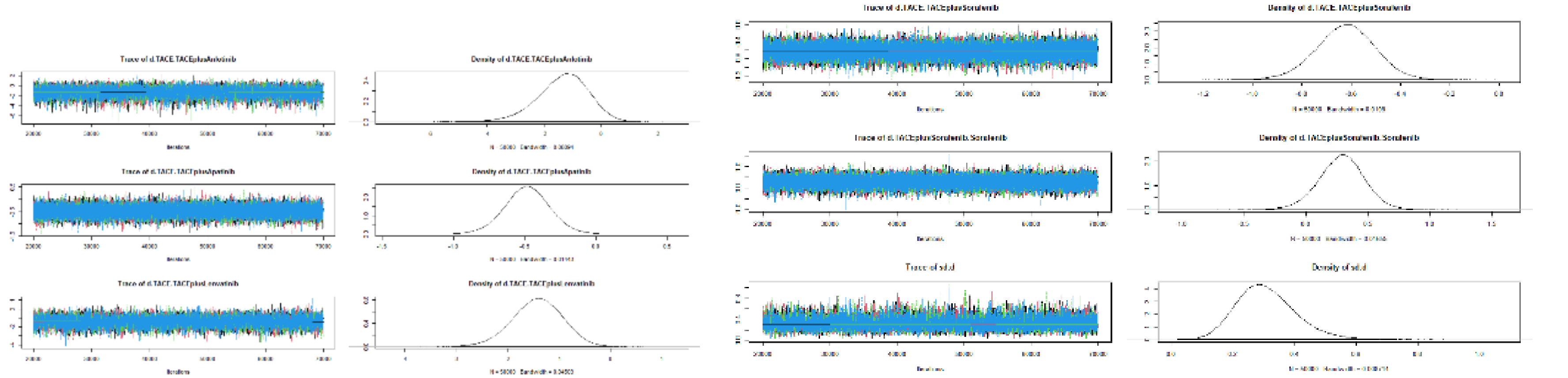
Trace of d.TACE.TACEplusAnlotinib
Density of d.TACE.TACEplusAnlotinib
Trace of d.TACE.TACEplusApatinib
Density of d.TACE.TACEplusApatinib
Trace of sd.d
Density of sd.d

ORR

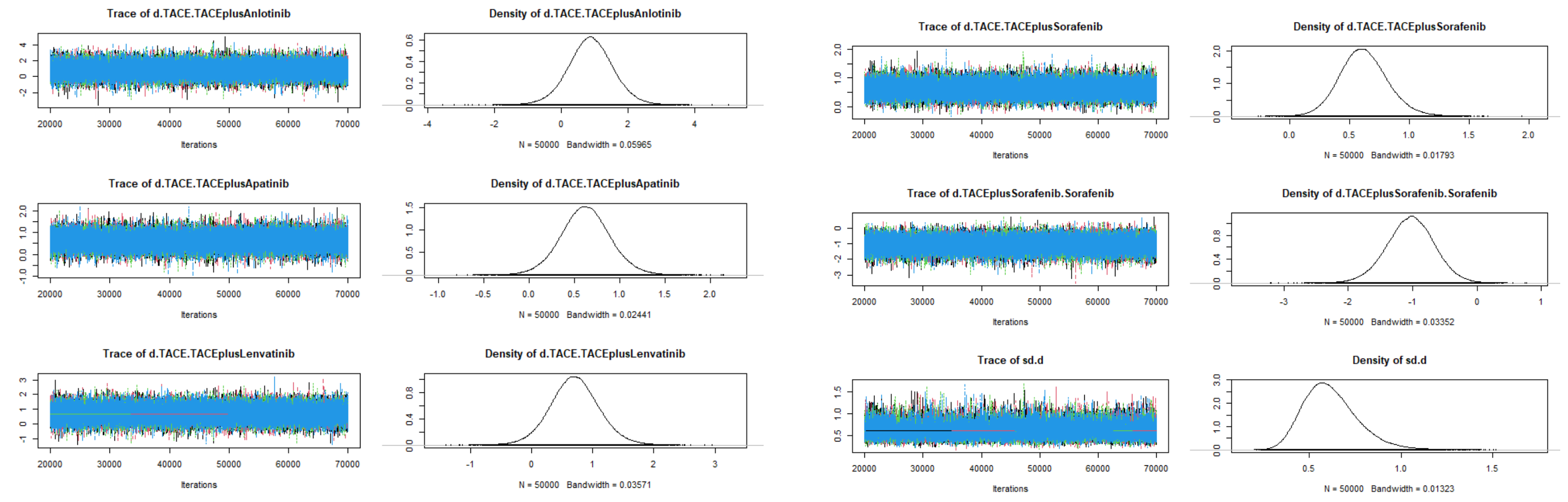
Trace of d.TACE.TACEplusAnlotinib
Density of d.TACE.TACEplusAnlotinib
N = 50000 Bandwidth = 0.05965
Trace of d.TACE.TACEplusApatinib
Density of d.TACE.TACEplusApatinib
N = 50000 Bandwidth = 0.02441
Trace of d.TACE.TACEplusLenvatinib
Density of d.TACE.TACEplusLenvatinib
N = 50000 Bandwidth = 0.03571
Trace of d.TACE.TACEplusSorafenib
Density of d.TACE.TACEplusSorafenib
N = 50000 Bandwidth = 0.01793
Trace of d.TACEplusSorafenib.Sorafenib
Density of d.TACEplusSorafenib.Sorafenib
N = 50000 Bandwidth = 0.03352
Trace of sd.d
Density of sd.d
N = 50000 Bandwidth = 0.01323

DCR

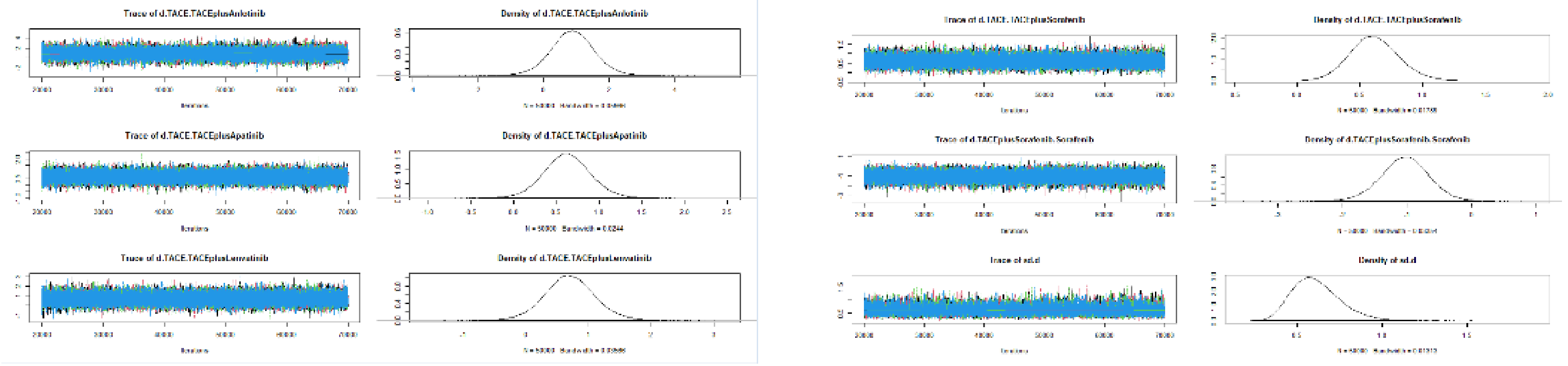
Trace of d.TACE.TACEplusAnlotinib
Density of d.TACE.TACEplusAnlotinib
Trace of d.TACE.TACEplusApatinib
Density of d.TACE.TACEplusApatinib
Trace of d.TACE.TACEplusLenvatinib
Density of d.TACE.TACEplusLenvatinib

Supplementary Material S7. Results of publication bias (the Funnel Plot of Enrolled Trials)

Note: “A” refers to " Sorfenib "; “B” refers to "TACE"; “C” refers to " TACE plus Anlotinib"; “D” refers to " TACE plus Apatinib"; “E” refers to " TACE plus Lenvatinib "; “F” refers to " TACE plus Sorafenib ".

OS

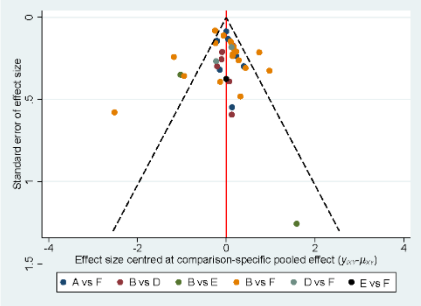

PFS

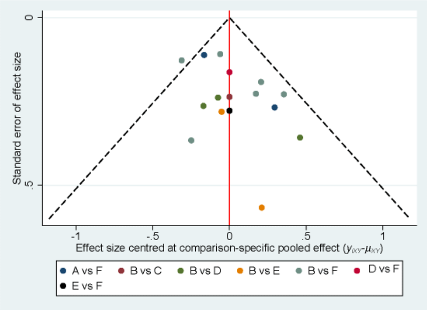

CR

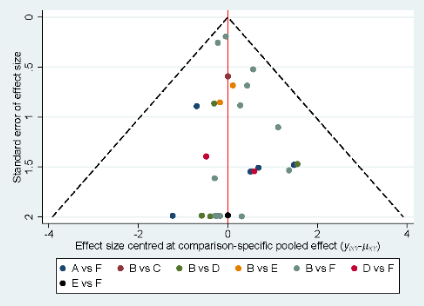

PR

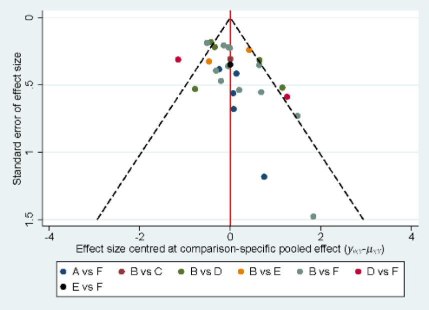

SD

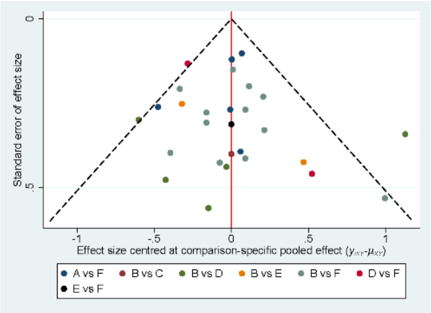

PD

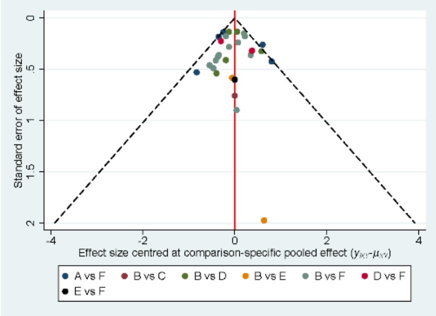

ORR

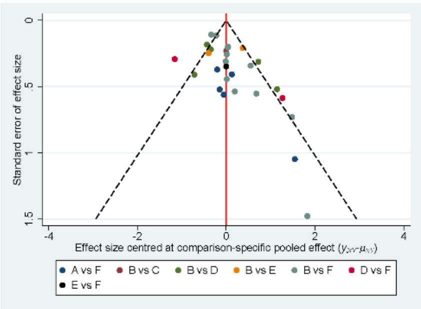

DCR

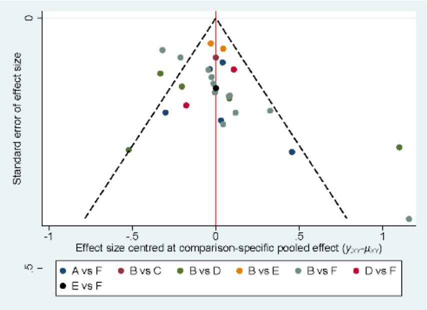

Supplementary Material S8. Node-splitting method for assessing consistency between direct and indirect evidence

A node-splitting method was used to check the consistency between direct and indirect evidence, and the geometry of the treatment network was established using R software.

OS

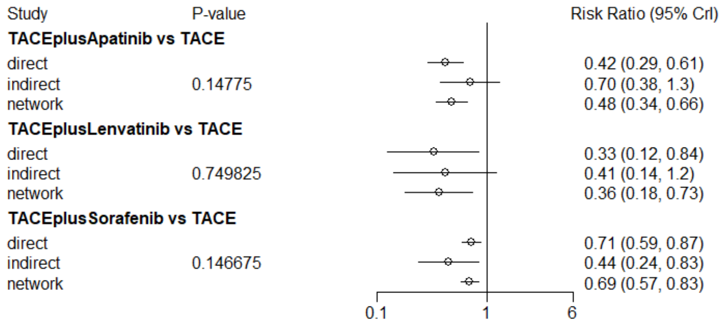

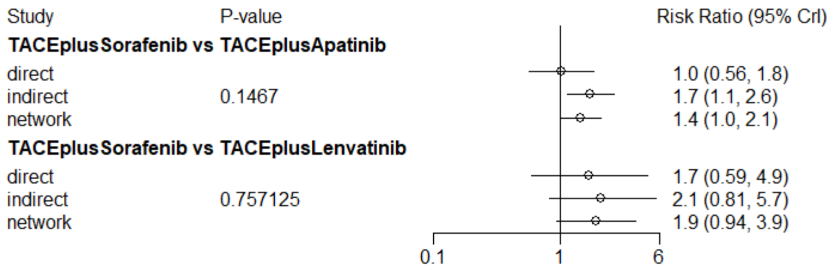

PFS

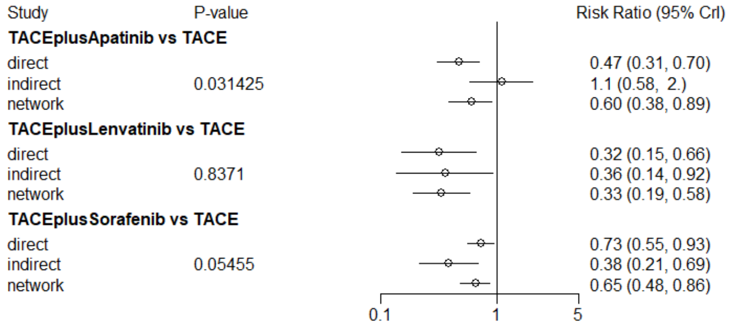

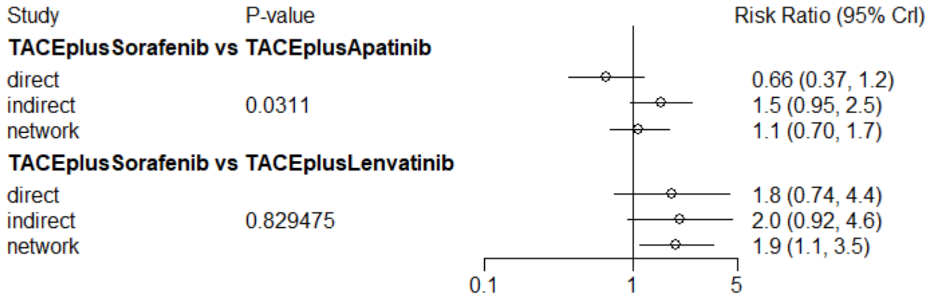

CR

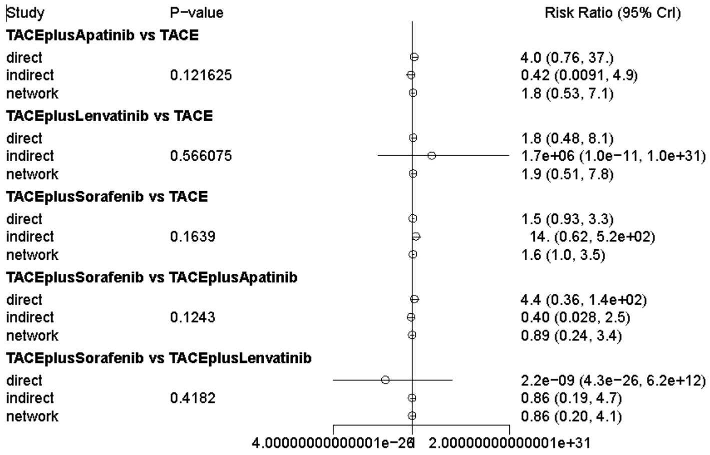
Study
P−value
Risk Ratio (95% CrI)
TACEplusApatinib vs TACE
direct
4.0 (0.76, 37.)
indirect
0.121625
0.42 (0.0091, 4.9)
network
1.8 (0.53, 7.1)
TACEplusLenvatinib vs TACE
direct
1.8 (0.48, 8.1)
indirect
0.566075
1.7e+06 (1.0e−11, 1.0e+31)
network
1.9 (0.51, 7.8)
TACEplusSorafenib vs TACE
direct
1.5 (0.93, 3.3)
indirect
0.1639
14. (0.62, 5.2e+02)
network
1.6 (1.0, 3.5)
TACEplusSorafenib vs TACEplusApatinib
direct
4.4 (0.36, 1.4e+02)
indirect
0.1243
0.40 (0.028, 2.5)
network
0.89 (0.24, 3.4)
TACEplusSorafenib vs TACEplusLenvatinib
direct
2.2e−09 (4.3e−26, 6.2e+12)
indirect
0.4182
0.86 (0.19, 4.7)
network
0.86 (0.20, 4.1)
4.00000000000001e−26
1
2.00000000000001e+31

PR

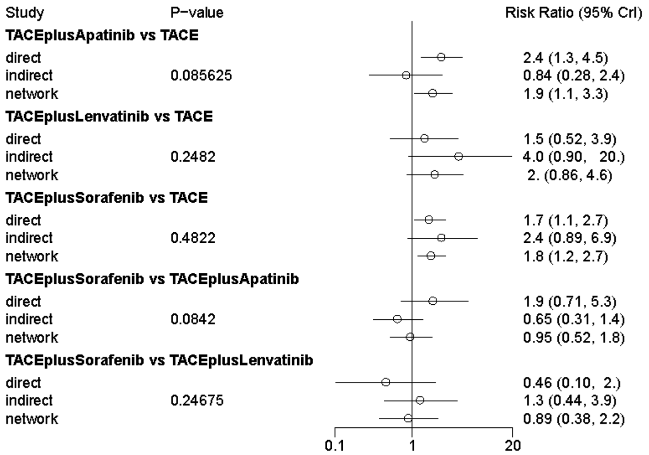
Study
P−value
Risk Ratio (95% CrI)
TACEplusApatinib vs TACE
direct
2.4 (1.3, 4.5)
indirect
0.085625
0.84 (0.28, 2.4)
network
1.9 (1.1, 3.3)
TACEplusLenvatinib vs TACE
direct
1.5 (0.52, 3.9)
indirect
0.2482
4.0 (0.90, 20.)
network
2. (0.86, 4.6)
TACEplusSorafenib vs TACE
direct
1.7 (1.1, 2.7)
indirect
0.4822
2.4 (0.89, 6.9)
network
1.8 (1.2, 2.7)
TACEplusSorafenib vs TACEplusApatinib
direct
1.9 (0.71, 5.3)
indirect
0.0842
0.65 (0.31, 1.4)
network
0.95 (0.52, 1.8)
TACEplusSorafenib vs TACEplusLenvatinib
direct
0.46 (0.10, 2.)
indirect
0.24675
1.3 (0.44, 3.9)
network
0.89 (0.38, 2.2)
0.1
1
20

SD

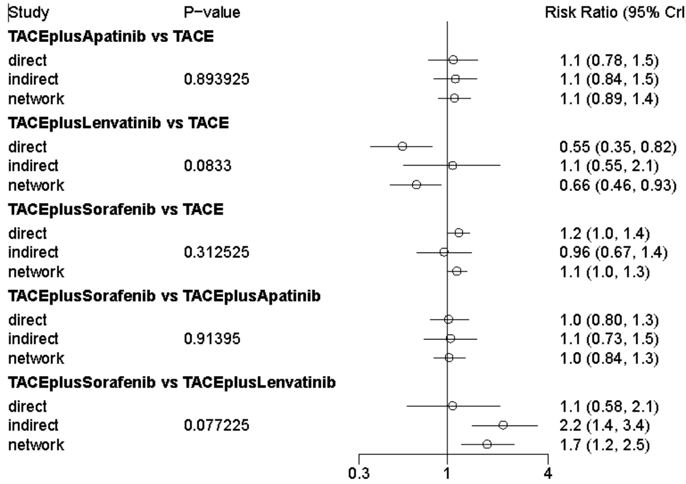
Study
P−value
Risk Ratio (95% CrI)
TACEplusApatinib vs TACE
direct
1.1 (0.78, 1.5)
indirect
0.893925
1.1 (0.84, 1.5)
network
1.1 (0.89, 1.4)
TACEplusLenvatinib vs TACE
direct
0.55 (0.35, 0.82)
indirect
0.0833
1.1 (0.55, 2.1)
network
0.66 (0.46, 0.93)
TACEplusSorafenib vs TACE
direct
1.2 (1.0, 1.4)
indirect
0.312525
0.96 (0.67, 1.4)
network
1.1 (1.0, 1.3)
TACEplusSorafenib vs TACEplusApatinib
direct
1.0 (0.80, 1.3)
indirect
0.91395
1.1 (0.73, 1.5)
network
1.0 (0.84, 1.3)
TACEplusSorafenib vs TACEplusLenvatinib
direct
1.1 (0.58, 2.1)
indirect
0.077225
2.2 (1.4, 3.4)
network
1.7 (1.2, 2.5)
0.3
1
4

PD

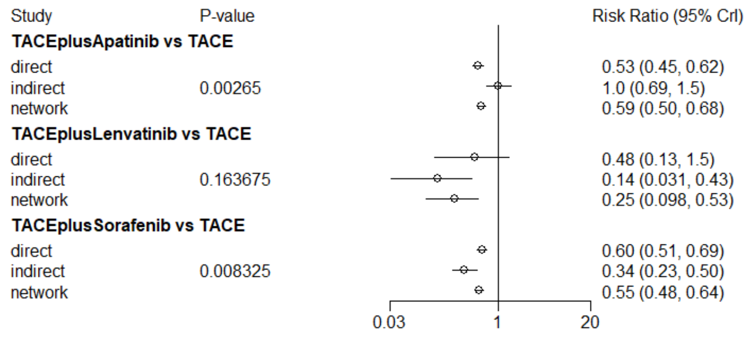

Study
P-value
Risk Ratio (95% CrI)
TACEplusApatinib vs TACE
direct
indirect
network
0.00265
0.53 (0.45, 0.62)
1.0 (0.69, 1.5)
0.59 (0.50, 0.68)
TACEplusLenvatinib vs TACE
direct
indirect
network
0.163675
0.48 (0.13, 1.5)
0.14 (0.031, 0.43)
0.25 (0.098, 0.53)
TACEplusSorafenib vs TACE
direct
indirect
network
0.008325
0.60 (0.51, 0.69)
0.34 (0.23, 0.50)
0.55 (0.48, 0.64)
0.03
1
20

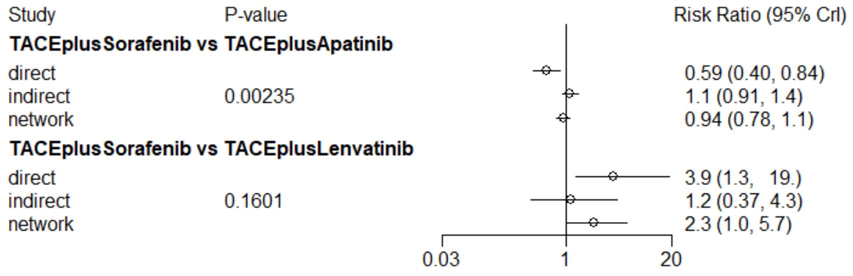

Study
P-value
Risk Ratio (95% CrI)
TACEplus Sorafenib vs TACEplusApatinib
direct
indirect
network
0.00235
0.59 (0.40, 0.84)
1.1 (0.91, 1.4)
0.94 (0.78, 1.1)
TACEplus Sorafenib vs TACEplusLenvatinib
direct
indirect
network
0.1601
3.9 (1.3, 19.)
1.2 (0.37, 4.3)
2.3 (1.0, 5.7)
0.03
1
20

ORR

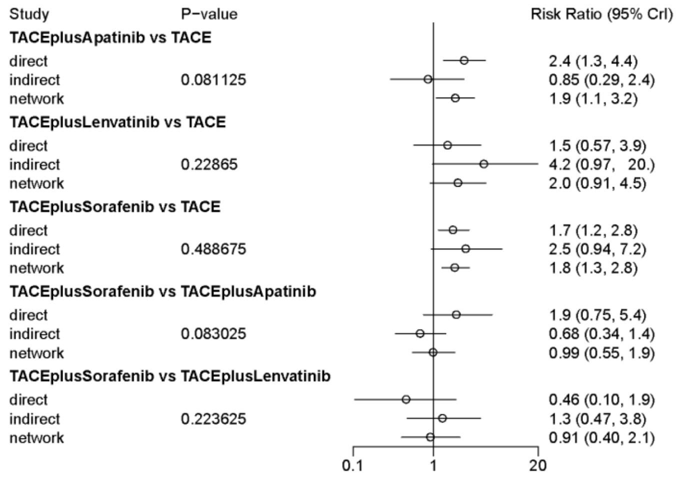

Study
P−value
Risk Ratio (95% CrI)
TACEplusApatinib vs TACE
direct
indirect
network
0.081125
2.4 (1.3, 4.4)
0.85 (0.29, 2.4)
1.9 (1.1, 3.2)
TACEplusLenvatinib vs TACE
direct
indirect
network
0.22865
1.5 (0.57, 3.9)
4.2 (0.97, 20.)
2.0 (0.91, 4.5)
TACEplusSorafenib vs TACE
direct
indirect
network
0.488675
1.7 (1.2, 2.8)
2.5 (0.94, 7.2)
1.8 (1.3, 2.8)
TACEplusSorafenib vs TACEplusApatinib
direct
indirect
network
0.083025
1.9 (0.75, 5.4)
0.68 (0.34, 1.4)
0.99 (0.55, 1.9)
TACEplusSorafenib vs TACEplusLenvatinib
direct
indirect
network
0.223625
0.46 (0.10, 1.9)
1.3 (0.47, 3.8)
0.91 (0.40, 2.1)
0.1
1
20

DCR

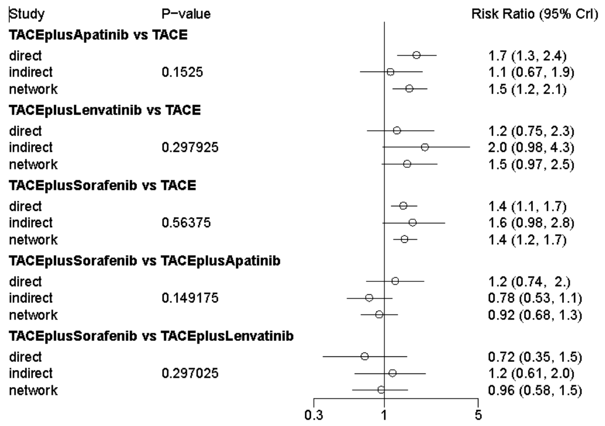

Study
P−value
Risk Ratio (95% CrI)
TACEplusApatinib vs TACE
direct
indirect
network
0.1525
1.7 (1.3, 2.4)
1.1 (0.67, 1.9)
1.5 (1.2, 2.1)
TACEplusLenvatinib vs TACE
direct
indirect
network
0.297925
1.2 (0.75, 2.3)
2.0 (0.98, 4.3)
1.5 (0.97, 2.5)
TACEplusSorafenib vs TACE
direct
indirect
network
0.56375
1.4 (1.1, 1.7)
1.6 (0.98, 2.8)
1.4 (1.2, 1.7)
TACEplusSorafenib vs TACEplusApatinib
direct
indirect
network
0.149175
1.2 (0.74, 2.)
0.78 (0.53, 1.1)
0.92 (0.68, 1.3)
TACEplusSorafenib vs TACEplusLenvatinib
direct
indirect
network
0.297025
0.72 (0.35, 1.5)
1.2 (0.61, 2.0)
0.96 (0.58, 1.5)
0.3
1
5

Supplementary Material S9. Forest plot of the outcomes.

OS

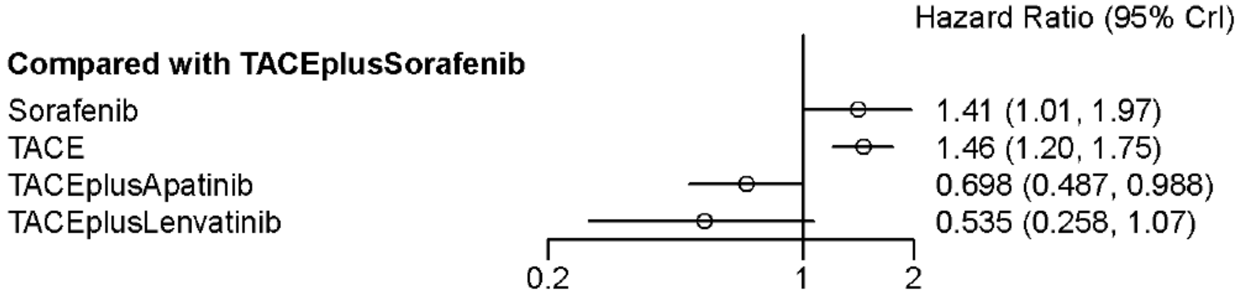

PFS

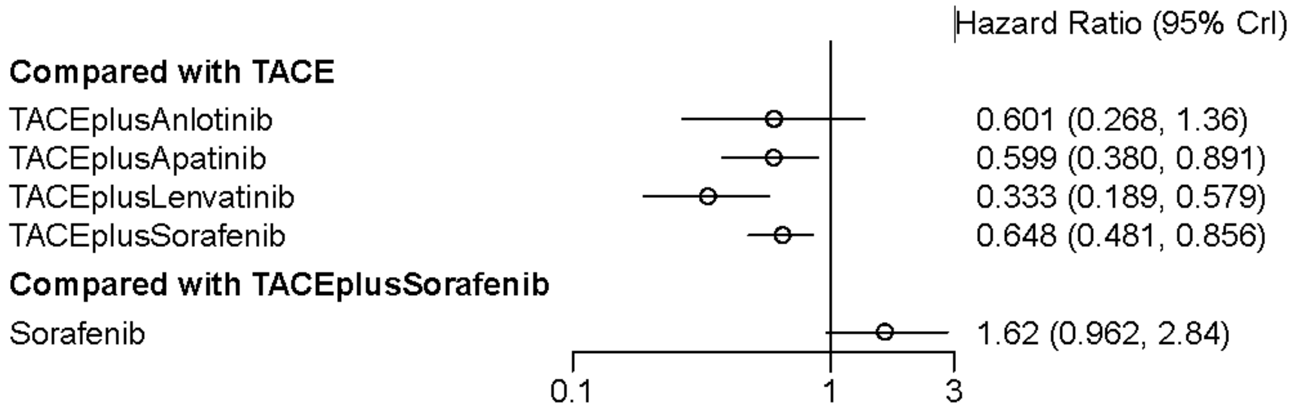

CR

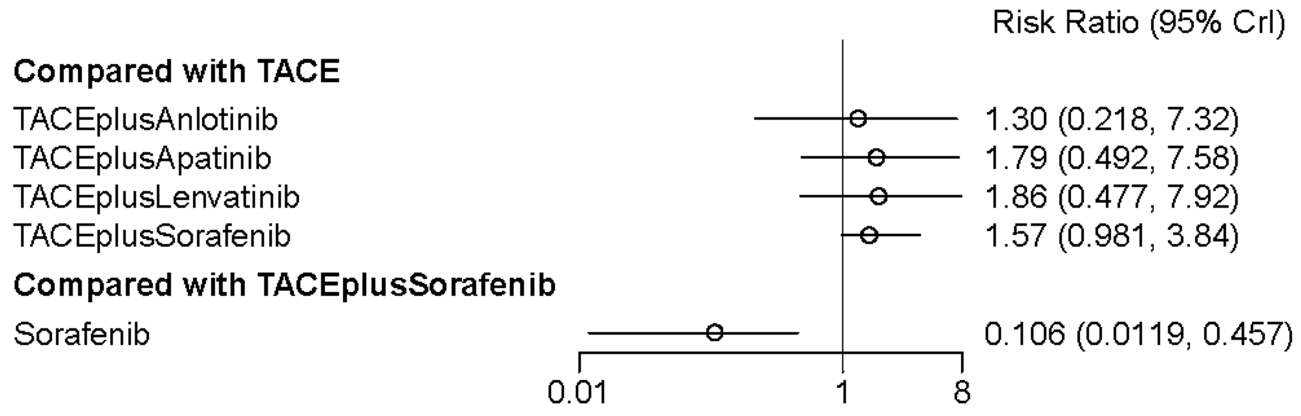
Risk Ratio (95% CrI)
Compared with TACE
TACEplusAnlotinib
1.30 (0.218, 7.32)
TACEplusApatinib
1.79 (0.492, 7.58)
TACEplusLenvatinib
1.86 (0.477, 7.92)
TACEplusSorafenib
1.57 (0.981, 3.84)
Compared with TACEplusSorafenib
Sorafenib
0.106 (0.0119, 0.457)
0.01
1
8

PR

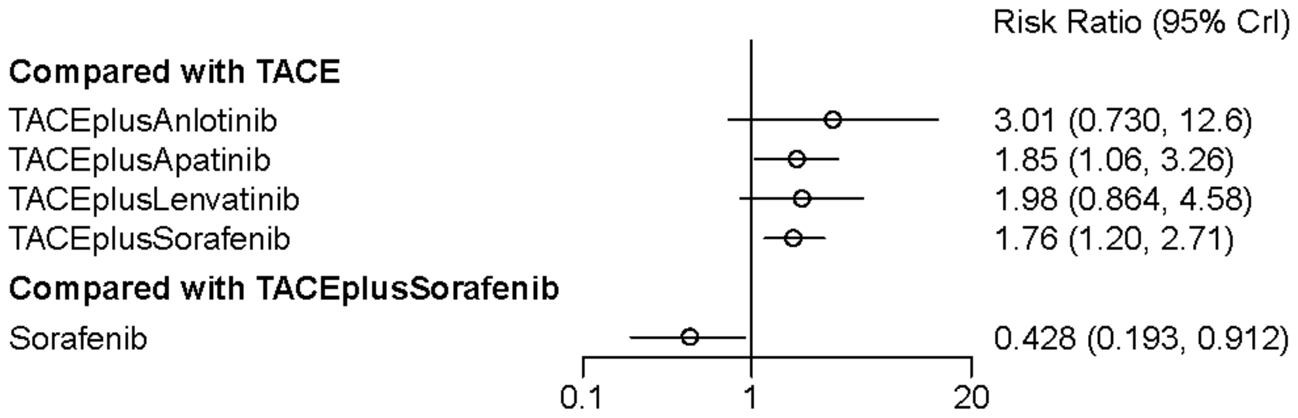
Risk Ratio (95% CrI)
Compared with TACE
TACEplusAnlotinib
3.01 (0.730, 12.6)
TACEplusApatinib
1.85 (1.06, 3.26)
TACEplusLenvatinib
1.98 (0.864, 4.58)
TACEplusSorafenib
1.76 (1.20, 2.71)
Compared with TACEplusSorafenib
Sorafenib
0.428 (0.193, 0.912)
0.1
1
20

SD

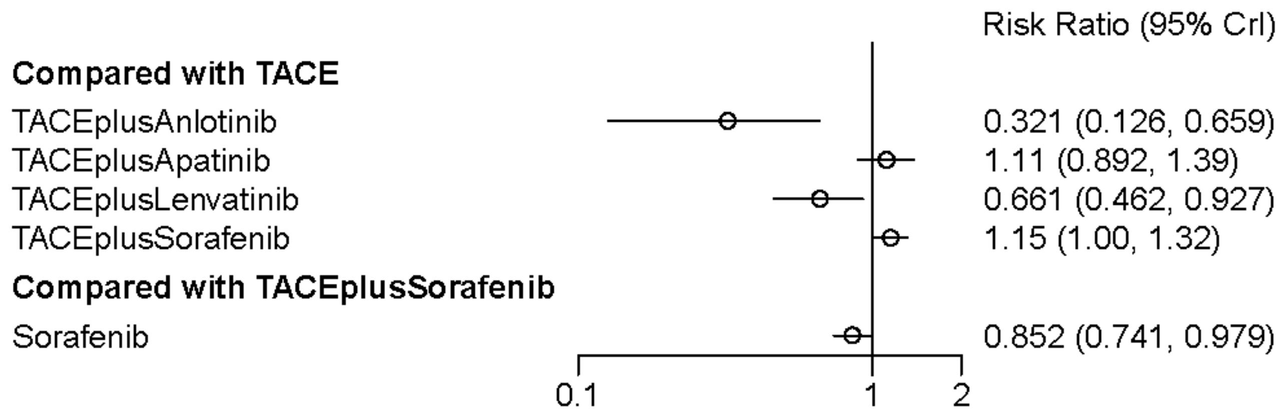
Risk Ratio (95% CrI)
Compared with TACE
TACEplusAnlotinib 0.321 (0.126, 0.659)
TACEplusApatinib 1.11 (0.892, 1.39)
TACEplusLenvatinib 0.661 (0.462, 0.927)
TACEplusSorafenib 1.15 (1.00, 1.32)
Compared with TACEplusSorafenib
Sorafenib 0.852 (0.741, 0.979)
0.1
1
2

PD

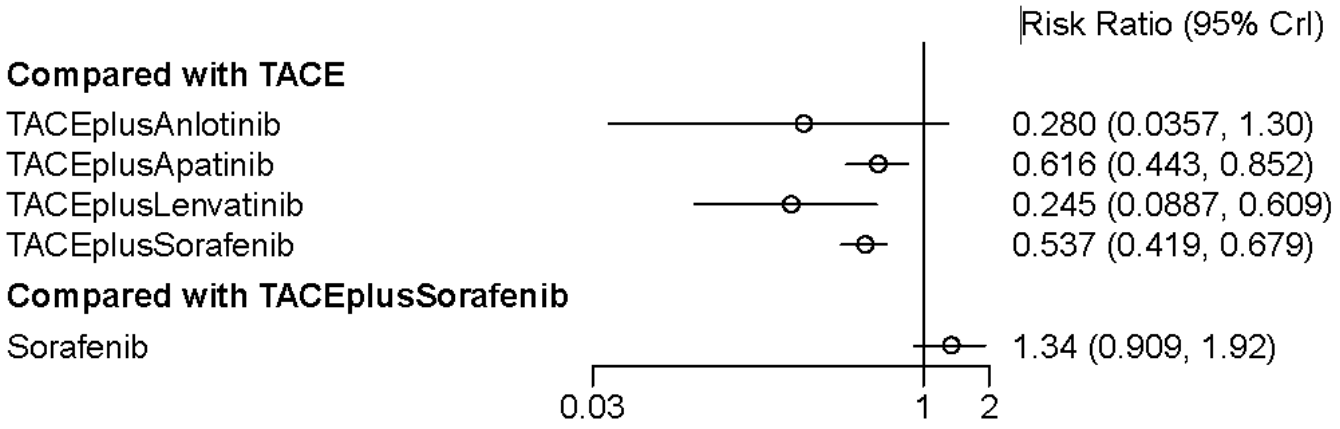
Risk Ratio (95% CrI)
Compared with TACE
TACEplusAnlotinib 0.280 (0.0357, 1.30)
TACEplusApatinib 0.616 (0.443, 0.852)
TACEplusLenvatinib 0.245 (0.0887, 0.609)
TACEplusSorafenib 0.537 (0.419, 0.679)
Compared with TACEplusSorafenib
Sorafenib 1.34 (0.909, 1.92)
0.03
1
2

ORR

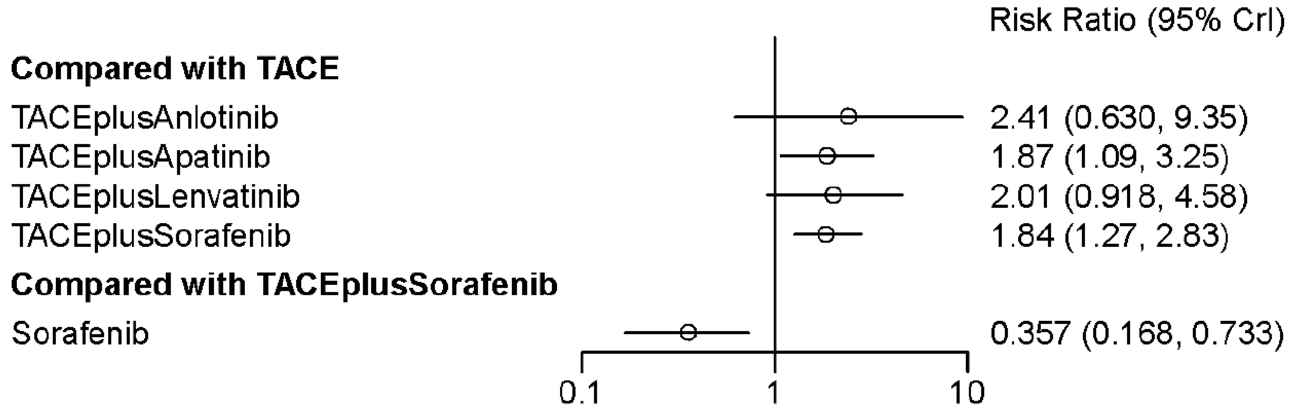
Risk Ratio (95% CrI)
Compared with TACE
TACEplusAnlotinib 2.41 (0.630, 9.35)
TACEplusApatinib 1.87 (1.09, 3.25)
TACEplusLenvatinib 2.01 (0.918, 4.58)
TACEplusSorafenib 1.84 (1.27, 2.83)
Compared with TACEplusSorafenib
Sorafenib 0.357 (0.168, 0.733)
0.1
1
10

DCR

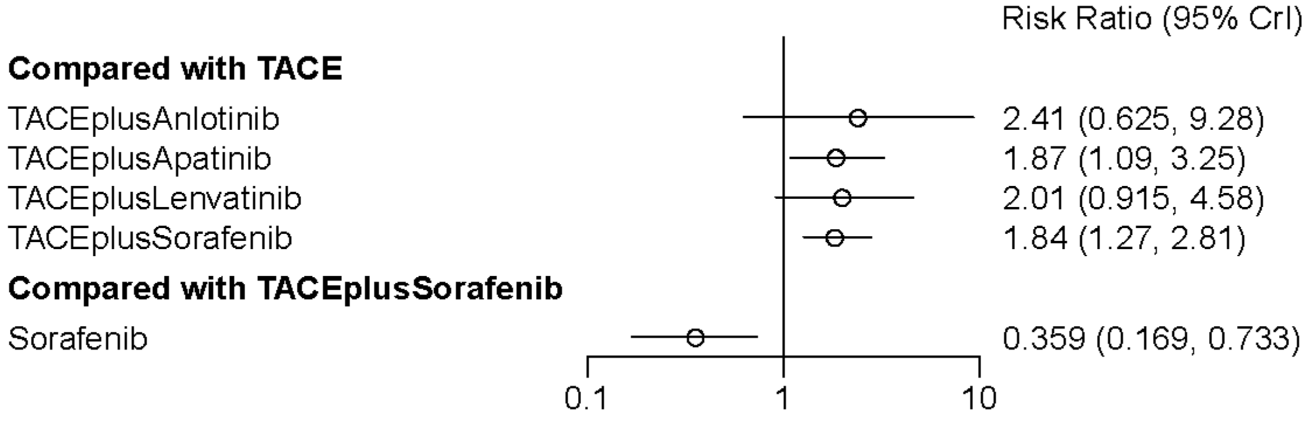
Risk Ratio (95% CrI)
Compared with TACE
TACEplusAnlotinib 2.41 (0.625, 9.28)
TACEplusApatinib 1.87 (1.09, 3.25)
TACEplusLenvatinib 2.01 (0.915, 4.58)
TACEplusSorafenib 1.84 (1.27, 2.81)
Compared with TACEplusSorafenib
Sorafenib 0.359 (0.169, 0.733)
0.1
1
10
